# Arousal modulates functional connectivity through structured and hemispherically asymmetric community architecture during wakefulness

**DOI:** 10.7554/eLife.110294

**Published:** 2026-06-24

**Authors:** Xiangyu Kong, Siyu Li, Gaolang Gong

**Affiliations:** 1 State Key Laboratory of Cognitive Neuroscience and Learning and IDG/McGovern Institute for Brain Research, Beijing Normal University Beijing China; 2 Beijing Key Laboratory of Brain Imaging and Connectomics, Beijing Normal University Beijing China; 3 Chinese Institute for Brain Research Beijing China; Monash University Australia; Radboud University Nijmegen Netherlands

**Keywords:** arousal, time-varying functional connectivity, hemispheric lateralization, resting fMRI, Human

## Abstract

Arousal fluctuates continuously during wakefulness, yet how these moment-to-moment variations shape large-scale functional connectivity (FC) remains unclear. Here, we combined 7T fMRI with concurrent pupillometry to quantify, for every functional connection, how time-varying FC covaries with spontaneous arousal in the awake human brain. Rather than exerting a uniform influence across the connectome, arousal organized FC into a low-dimensional set of seven connectivity communities, each defined by characteristic network compositions. These communities exhibited systematic hemispheric asymmetries, specifically identifying a ‘left-hemisphere centripetal architecture’ where the left hemisphere serves as a structural sink for the asymmetric convergence of arousal-modulated signals. Importantly, hemispheric asymmetry did not arise from global shifts in connectivity strength but instead reflected structured spatial heterogeneity embedded within community architecture. This modular and asymmetric organization was highly preserved during naturalistic movie watching, indicating that arousal-related modulation of FC reflects intrinsic principles that generalize across awake cognitive contexts. Together, these findings demonstrate that moment-to-moment arousal fluctuations shape large-scale FC through structured, hemispherically asymmetric network organization during wakefulness.

## Introduction

Arousal is a core dimension of brain state that shapes perception, attention, and the coordination of large-scale neural systems. Across major transitions—such as wakefulness to sleep, anesthesia, or disorders of consciousness—substantial reorganization of functional connectivity (FC) has been consistently observed (Chow et al., 2013; Tagliazucchi et al., 2016; Demertzi et al., 2019; Banks et al., 2020; Damaraju et al., 2020; Huang et al., 2020; Jang et al., 2024). These state-based findings demonstrate that arousal fundamentally influences whole-brain communication patterns. However, such approaches typically contrast discrete arousal states and, therefore, provide limited insight into how moment-to-moment fluctuations in arousal within the awake brain influence the organization of functional connectivity.

Increasing evidence shows that arousal varies continuously even during stable wakefulness, including during resting-state fMRI and ongoing cognitive engagement. These spontaneous fluctuations, often indexed by pupil diameter (Reimer et al., 2014; McGinley et al., 2015; Joshi and Gold, 2020), modulate neural gain, sensory responses, and behavioral performance. Yet despite their ubiquity and behavioral relevance, it remains largely unknown how fine-grained arousal variations are expressed across the functional connectome. Prior work has primarily examined regional signal amplitude or isolated networks (Yellin et al., 2015; Schneider et al., 2016; Breeden et al., 2017; Podvalny et al., 2021; Sobczak et al., 2021; Lloyd et al., 2023), thereby leaving unresolved the fundamental question of whether the awake brain’s FC is uniformly sensitive to arousal or whether arousal instead imprints structured spatial patterns across functional networks.

A parallel question is whether arousal-modulated connectivity patterns show hemispheric asymmetry, given longstanding evidence for lateralized arousal, vigilance, and alerting mechanisms. Studies of unihemispheric sleep and lateralized arousal dynamics in animals (Rattenborg et al., 2000; Lyamin et al., 2016; Mascetti, 2016; Reicher et al., 2021; Fenk et al., 2023; Libourel et al., 2023), asymmetries in human EEG-based vigilance (Tamaki et al., 2016), and right-lateralized alerting functions in attention (Heilman and Van Den Abell, 1980; Sturm and Willmes, 2001; Shulman et al., 2010; Corbetta and Shulman, 2011) all suggest that arousal may modulate left and right hemispheric systems differently. Nevertheless, these observations have yet to be linked to the organization of whole-brain functional interactions, and it remains unknown whether such asymmetries manifest at the level of large-scale FC, and whether they reflect organized connectivity patterns rather than non-specific global effects.

To address these questions, we combined high-field fMRI with concurrent pupillometry to quantify, for every functional connection, how its connectivity covaries with spontaneous arousal fluctuations during wakefulness. This edgewise measure of arousal–time varying functional connectivity (tvFC) coupling provides a comprehensive map of where in the connectome arousal leaves its strongest imprint, without imposing predefined states or regional assumptions. Using this framework, we first test whether arousal sensitivity is spatially homogeneous or segregates into distinct sets of connections with similar coupling profiles. We next assess whether these spatially organized arousal-modulated patterns show systematic hemispheric asymmetry, with particular emphasis on attentional systems that show known lateralization. Finally, we evaluate the cross-context stability of this organizational structure by comparing resting state and naturalistic movie watching in the same participants. Together, these analyses delineate how moment-to-moment arousal fluctuations shape large-scale functional architecture in the awake human brain.

## Results

**Figure 1.**
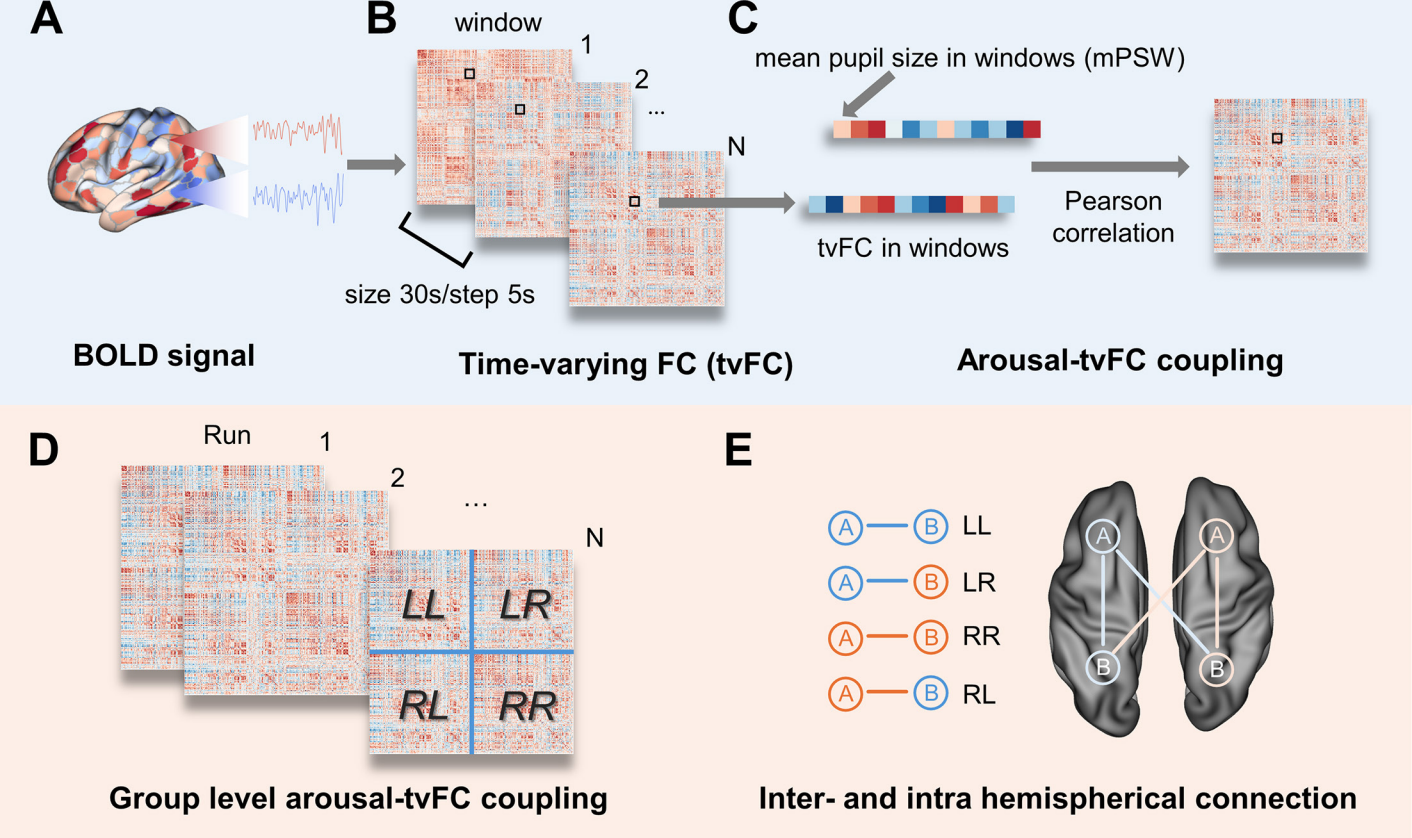
Pipeline for estimating arousal–time varying functional connectivity (tvFC) coupling from fMRI and pupillometry. (**A**) Concurrent 7T fMRI and eye tracking were collected during resting state and naturalistic movie watching. (**B**) tvFC was computed over sliding windows for each pair of brain regions. (**C**) Pupil diameter was preprocessed to obtain a continuous arousal time series. Arousal–tvFC coupling for each connection was then defined as the Pearson correlation between its windowed functional connectivity (FC) time course and the corresponding arousal fluctuations. (**D**) These edgewise coupling values yielded a dense arousal–tvFC coupling matrix per run, which served as the input for analyses of community structure, hemispheric asymmetry, and cross-paradigm consistency. (**E**) The connections were categorized based on the hemispheres of the connected regions: Left-Left (LL) and Right-Right (RR) represent intra-hemispheric connections; Left-Right (LR) and Right-Left (RL) represent inter-hemispheric connections. This classification enabled hemisphere-specific analyses of arousal-tvFC coupling.

The processing procedure of estimating arousal–tvFC coupling from fMRI and pupillometry was illustrated in Figure 1. Here, we use the term arousal–tvFC coupling to refer to the regression-based estimate of how spontaneous arousal fluctuations modulate each functional connection over time.

While our primary inferential analyses were conducted at the run level to leverage the high-density sampling of the HCP 7T dataset, we further validated the robustness of these findings using participant-level statistical summaries and resampling to account for within-participant dependencies (see Figure 2—figure supplement 1, Figure 3—figure supplement 1, Figure 4—figure supplement 1 and Figure 5—figure supplement 1).

### Arousal–tvFC coupling reveals seven distinct connectivity communities

For each functional connection, we quantified how strongly its time-varying connectivity covaried with moment-to-moment arousal, producing an edgewise arousal–tvFC coupling matrix per run. Across all participants and runs, these coupling profiles showed clear structure: connections did not exhibit uniform arousal sensitivity but instead formed separable groups.

**Figure 2.**
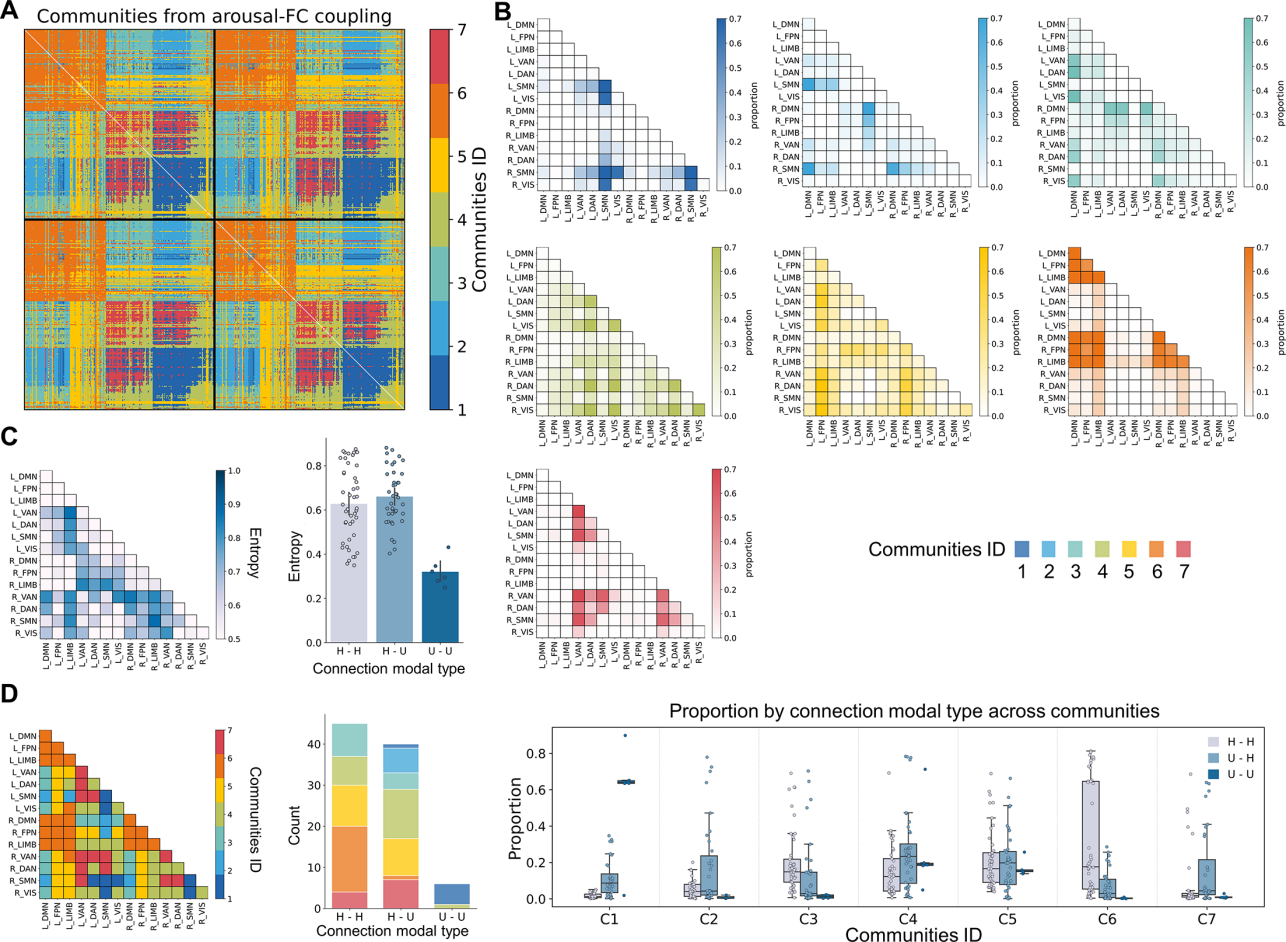
Arousal–time varying functional connectivity (tvFC) coupling partitions the connectome into seven distinct connectivity communities. (**A**) Unsupervised clustering of edgewise arousal–tvFC coupling identified seven stable communities, demonstrating that arousal-linked modulation is organized into low-dimensional structure rather than uniformly distributed across connections. (**B**) Mapping communities onto network-pair space revealed distinct and reproducible composition profiles (upper panel), with some communities dominated by heteromodal interactions and others enriched in H–U or U–U network pairs (bottom panel). (**C**) Community participation entropy varied systematically across connection modal types: U–U network pairs showed lower entropy, indicating concentrated engagement in a small subset of communities, whereas H-H and H–U network pairs exhibited significantly higher entropy (F(2,88)=12.24, *p*=4.38×10^–6^), reflecting broader distribution across communities. (**D**) Dominant-community assignments confirmed this organization, showing that heteromodal interactions load onto multiple arousal-sensitive communities, whereas U–U network pairs show more restricted community involvement.

Unsupervised clustering of edgewise coupling patterns identified seven stable connectivity communities (Figure 2A). This solution was consistently favored across a broad range of cluster numbers, indicating that arousal–tvFC coupling is inherently low-dimensional. The seven communities captured distinct patterns of arousal sensitivity across the connectome. Projecting each community into canonical network pairs space (Thomas Yeo et al., 2011) showed that these communities were not random mixtures of connections. Instead, each displayed a characteristic distribution across network pairs (Figure 2B). Some communities were enriched in network pairs linking heteromodal and unimodal systems (Mesulam, 1998), while others were dominated by heteromodal–heteromodal (H-H) or unimodal–unimodal (U-U) network pairs. Community participation entropy further highlighted this structure: unimodal–unimodal connections showed low entropy, indicating a restricted participation in a few communities, whereas H-H and heteromodal–unimodal (H-U) connections showed significantly higher entropy, indicating more diverse engagement across communities (F(2,88)=12.24, *p*=4.38×10^–6^).

**Figure 2—figure supplement 1.**
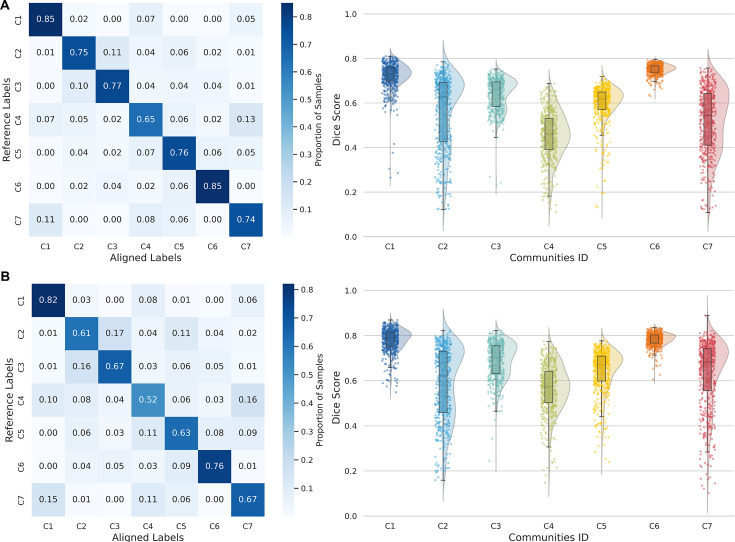
Reliability of community architecture across different resampling strategies. (**A**) Confusion matrix (left) showing the consistency between community labels from 500 iterations (aligned via the Hungarian algorithm) and the primary results. The right panel displays the distribution of Dice coefficients for each individual community label. (**B**) Stability of community architecture using participant-level resampling, following the same analytical framework and visualization as in (**A**).

Together, these results demonstrate that arousal does not uniformly modulate the connectome but instead engages a small number of organized connectivity communities, each with distinct network-level compositions.

The robustness of the seven-community architecture was cross-validated using both split-half and participant-level resampling strategies. As shown in Figure 2—figure supplement 1, both approaches yielded high alignment accuracy and consistently high Dice coefficients across all communities, confirming that the identified clusters are not artifacts of specific data partitioning.

### Arousal-modulated community architecture exhibit systematic hemispheric asymmetry

We next asked whether these arousal-modulated communities express hemispheric biases. Using integration and segregation indices derived from LL, RR, LR, and RL edge categories, we quantified, for each community, whether arousal preferentially modulated intra- versus inter-hemispheric connectivity and whether these effects favored one hemisphere.

**Figure 3.**
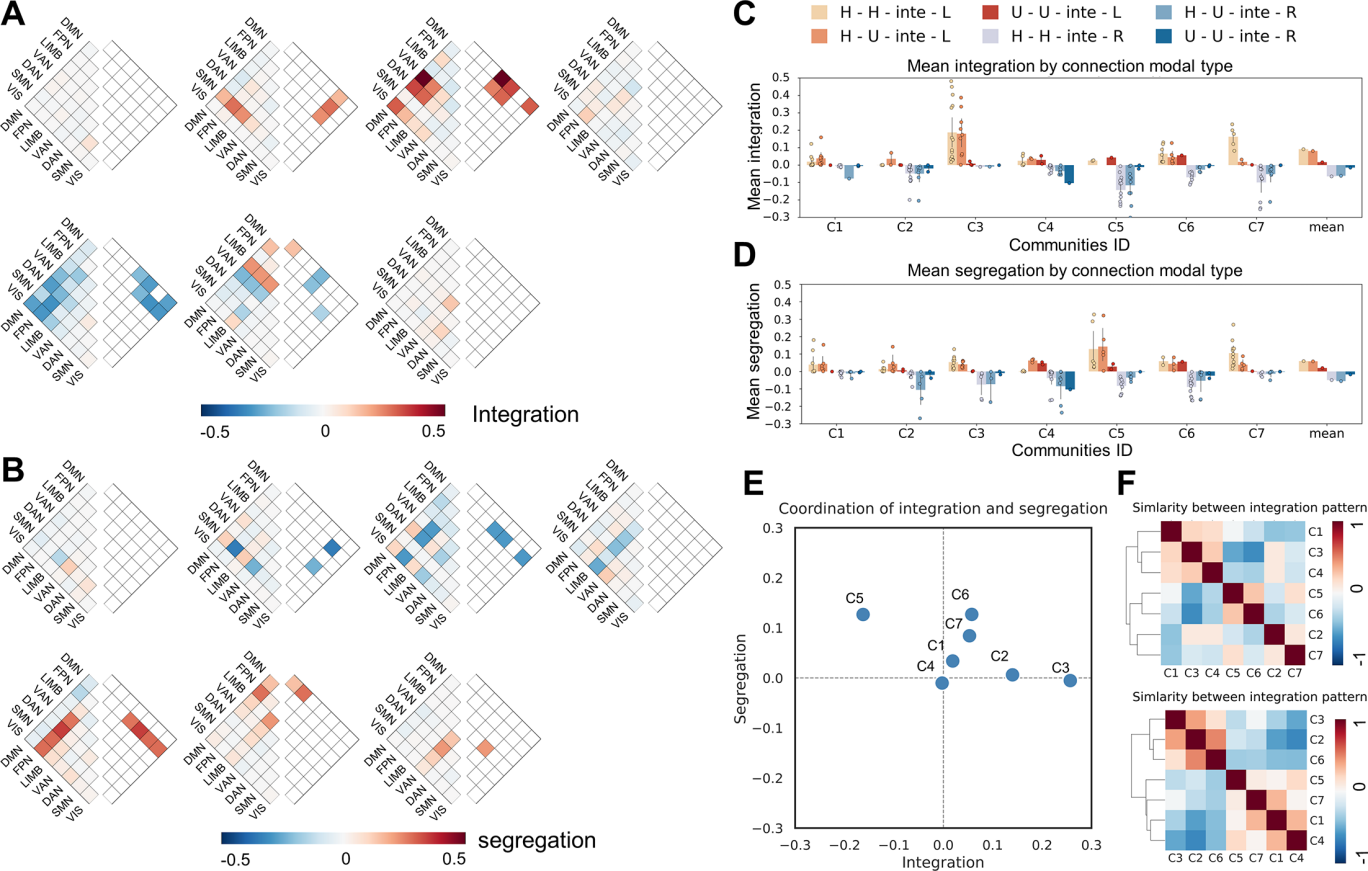
Arousal-modulated communities architecture exhibit community-specific hemispheric asymmetry. (**A**) Integration indices for each network pair revealed significant leftward or rightward deviations from a spatial permutation null, indicating that arousal differentially modulates between- and within-hemisphere interactions for specific network pairs. (**B**) Segregation indices identified network pairs showing hemisphere-specific strengthening of within-hemisphere connectivity, further demonstrating that lateralization is localized rather than global. (**C–D**) Community-averaged integration and segregation values showed that hemispheric biases vary across communities and are not determined solely by connection modal type, underscoring the community-specific nature of the asymmetry. (**E**) Aggregating all significantly lateralized network pairs, each community exhibited a distinct integration–segregation profile, revealing unique hemispheric signatures across communities. (**F**) Low similarity among communities’ integration and segregation patterns confirmed that arousal imposes multiple, community-specific forms of hemispheric asymmetry, rather than a single unified left- or right-dominant pattern.

At the network-pair level, several pairs showed significant lateralization compared with a spatial permutation null model (Figure 3A–B). Importantly, lateralization was not global: only specific network pairs within communities exhibited robust hemispheric biases, while others remained symmetric. Some communities showed rightward integration, indicating stronger arousal-related modulation of network pairs within the right hemisphere or between the right hemisphere and the rest of the brain, whereas others showed leftward segregation, reflecting preferential influence on within-left-hemisphere interactions.

**Figure 3—figure supplement 1.**
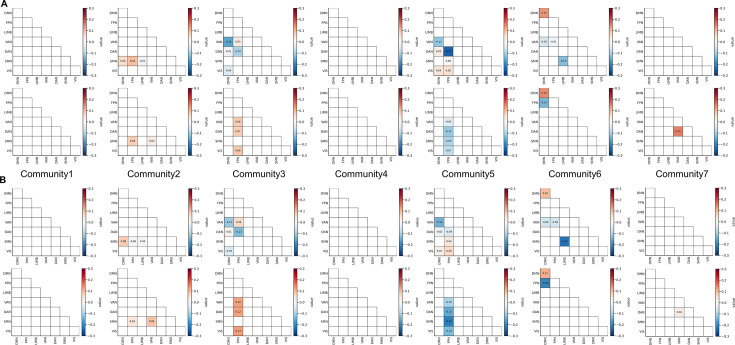
Reliability of network-pair specific hemispheric asymmetry in community architecture across resampling strategies. (**A**) The relative value was calculated as:,$\left (LI_{iter}- LI_{orig}\right)/LI_{orig}$ where $LI_{iter}$ represents the mean of 500 resampling iterations and，$LI_{orig}$ denotes the original findings. The top and bottom rows represent integration and segregation patterns, respectively. (**B**) Stability assessment using participant-level resampling, following the same calculation and visualization framework as in (**A**).

Averaging indices across connection modal types reinforced this community-specific patterning (Figure 3C–D). Notably, identical connection modal types (e.g. H-H) could show leftward bias in one community but rightward bias in another, demonstrating that lateralization is tied to the community architecture rather than to the canonical network pair class. Aggregating all significantly lateralized network pairs, each community expressed a unique lateralization signature (Figure 3E), and there was no clear cluster of lateralization patterns among communities (Figure 3F), indicating heterogeneous, rather than unified, hemispheric influences of arousal on FC. Instead, arousal imprints distinct left–right biases across different communities.

The robustness of the hemispheric lateralization of community architecture was further supported by the aforementioned resampling validations (Figure 3—figure supplement 1). For the mean integration and segregation indices across network pairs, the empirical values consistently fell within the center of the resampling distributions. Specifically, for the network-pair specific lateralization signatures, the directional biases observed in each community remained stable during resampling, with empirical values largely aligned with the center of their respective distributions. This high degree of consistency confirms that the reported community-specific asymmetry is a stable and representative feature of arousal-modulated organization, ensuring that our findings are not skewed by specific sample compositions or outliers.

### Gradients of community affiliation entropy and the centripetal lateralization architecture of community affiliation

**Figure 4.**
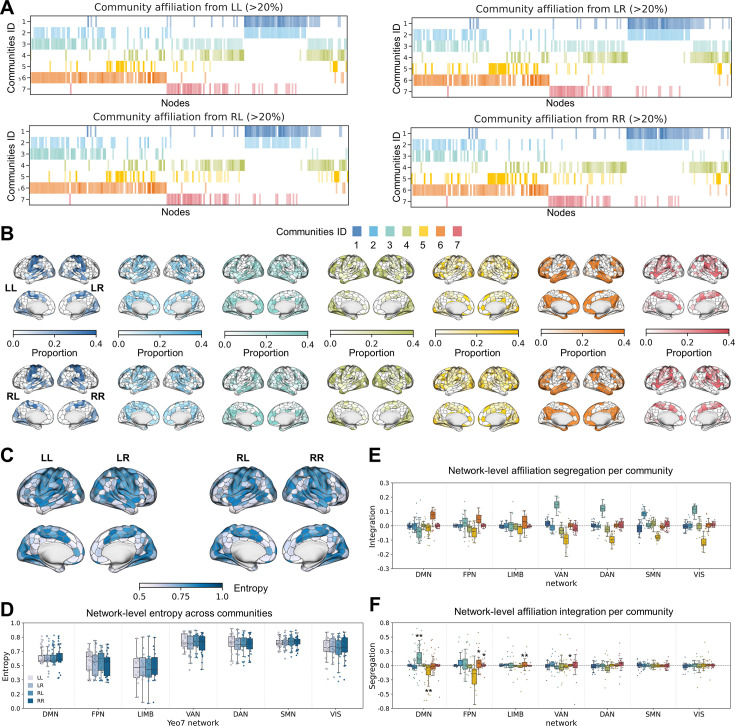
Characterizing the spatial distribution, entropy, and hemispheric divergence of regional community affiliation. (**A–B**) Nodal-level community affiliation matrices, computed separately for Left-Left (LL), Left-Right (LR), Right-Left (RL), and Right-Right (RR) edges, showed substantial heterogeneity in how nodes distribute their arousal–time varying functional connectivity (tvFC) coupling across the seven communities, with distinct patterns emerging across canonical networks. For visualization purposes, panel A is restricted to displaying values where the proportion exceeds 0.2. (**C–D**) Region-level community affiliation entropy revealed a systematic network gradient, in which heteromodal systems displayed more selective participation, whereas unimodal networks showed broader, more distributed engagement across communities (t(798) = –23.81, *p*=4.24×10^⁻95^) (**E**) No integration bias was detected in any community. (**F**) Significant leftward segregation biases were identified within specific communities (communities 3, 5, 6, and 7). These asymmetries were primarily localized in regions belonging to the DMN, FPN, LIMB, and VAN. The color of each box corresponds to the community identity. Statistical significance: * p_FDR <0.01; ** p_FDR <0.001.

Having established that arousal-modulated functional connections can be organized into modular communities with distinct hemispheric lateralization at the network level, we next explored the nodal-level mapping of these communities and the substantial variability in how regions participate in arousal-modulated communities. For each region, we computed its community affiliation for each brain region, defined as the proportion of edges connected to that region that were assigned to each of the seven communities, separately for LL, LR, RL, and RR edges (Figure 4A–B).

**Figure 4—figure supplement 1.**
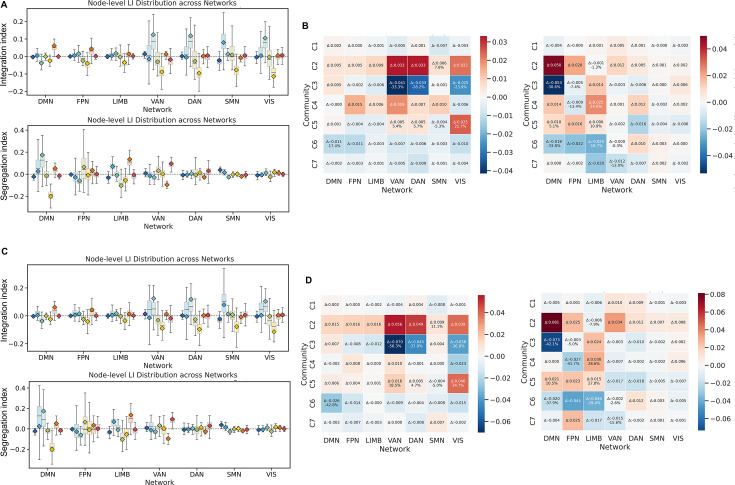
Reliability of regional affiliation asymmetry across resampling strategies. (**A**) Stability analysis using split-half resampling. Boxplots represent the distribution of asymmetry indices across 500 iterations, where colored diamonds indicate the original findings. (**B**) The heatmap background represents the absolute deviation (Δ) between the discovery sample and the resampled mean. Numerical annotations indicate the relative error percentage (%). To prevent the statistical inflation of relative values in regions with minimal effects, these annotations are explicitly omitted where the $LI_{orig}$ is low (<0.05), focusing the assessment on robustly lateralized regions. (**C–D**) Stability assessment using participant-level resampling, following the same quantitative framework. The overall tight alignment between resampled distributions and original discovery values confirms that the spatial patterns of regional hemispheric bias are highly stable and not artifacts of specific sample selection.

Next, we investigated the nodal-level community participation flexibility by calculating the community affiliation entropy for each region. Nodal-level affiliation entropy showed a clear network gradient (Figure 4C–D). Default modal network (DMN), frontoparietal network (FPN), and limbic (LIMB) regions exhibited lower entropy, indicating focused participation in fewer arousal-modulated communities. In contrast, dorsal attention network (DAN), somatomotor network (SMN), and visual (VIS) regions showed broader participation across communities (t(798) = –23.81, *p*=4.24×10⁻^95^).

To evaluate lateralization at the nodal level, we derived metrics of segregation and integration based on these affiliation profiles. We observed a common organizational principle across several key communities: while no significant hemispheric bias in integration was detected in any community (Figure 4E), Communities 3, 5, 6, and 7 exhibited robust leftward segregation biases (Figure 4F). This dissociation between segregation and integration reveals a pronounced centripetal lateralization architecture. A detailed examination of the regional affiliation profiles indicated a consistent directional bias in signal flow across these communities: whereas left-hemisphere projections were predominantly intra-hemispheric (LL >LR), right-hemisphere inputs were biased contralaterally toward the left side (RL >RR). These findings suggest that these communities collectively represent a ‘left-hemisphere centripetal architecture,’ where the left hemisphere serves as a preferential convergence of arousal-modulated signals, preferentially aggregating both ipsilateral and contralateral inputs.

The robustness of these observations was further supported by the aforementioned resampling validations (Figure 4—figure supplement 1). Regarding the hemispheric lateralization of affiliation profiles, the directional biases observed across regions and communities remained highly stable during resampling, with empirical values consistently aligned with the center of their respective distributions. This high degree of consistency confirms that the reported centripetal affiliation architecture is a stable and representative feature of arousal-modulated lateralization, ensuring that our findings are not skewed by specific sample compositions or outliers.

### Arousal-tvFC coupling lateralization arises from spatial heterogeneity rather than mean shifts

In the preceding analyses, we mainly focused on the lateralization of the organizational patterns of the decomposed communities at the network-pairs and nodal levels. However, how the strength of the arousal–tvFC coupling is spatially distributed and whether lateralization exists in this distribution remains elusive. We, therefore, next examined the spatial distribution of arousal–tvFC coupling strength and its lateralization properties.

To test whether hemispheric biases reflected simple shifts in mean arousal–tvFC coupling strength, we compared mean integration and segregation across communities and whole connectome. Although the inter-community difference for segregation was statistically significant, the mean difference between communities was very small. This indicates a limited hemispheric imbalance.

**Figure 5.**
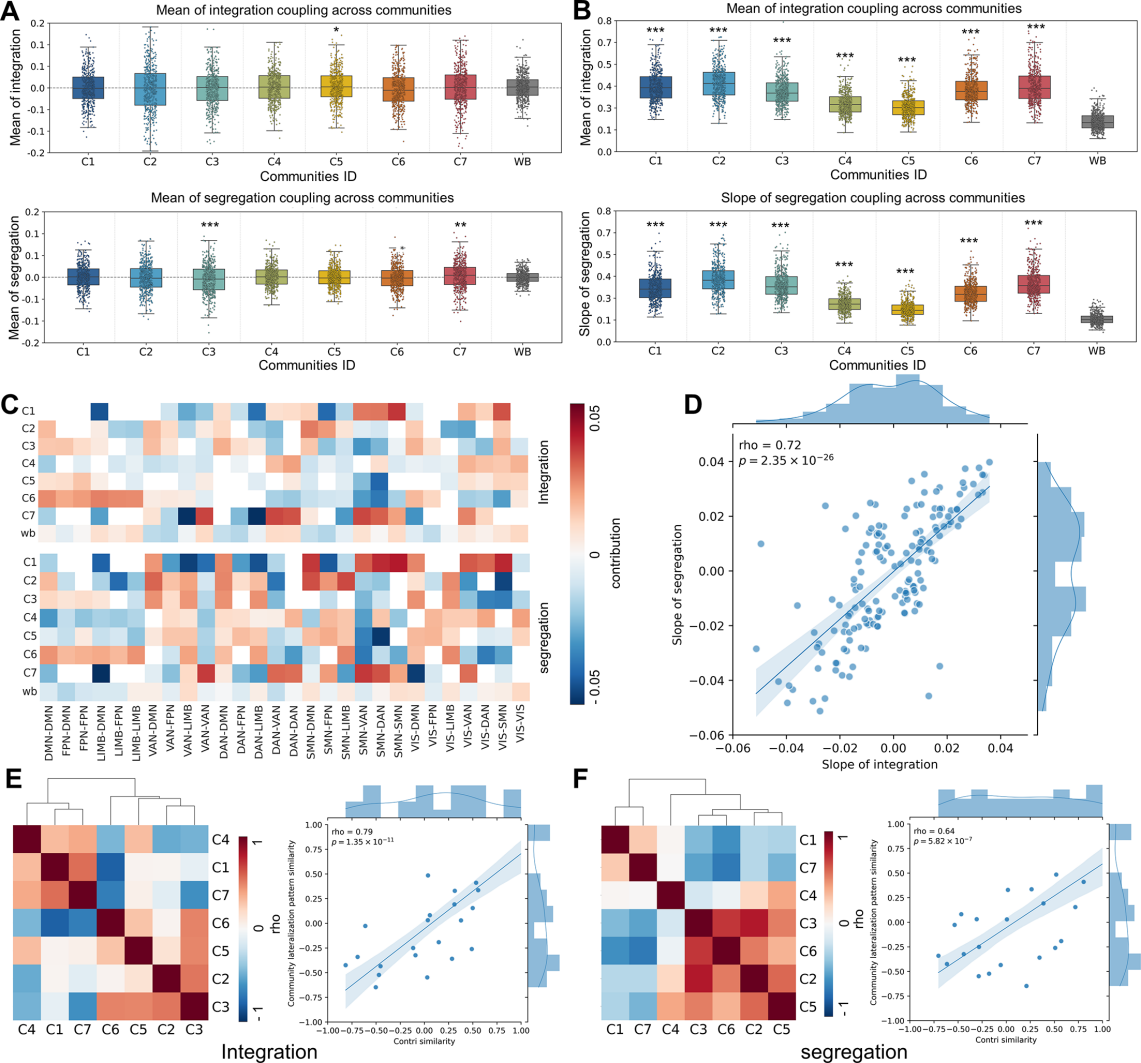
Spatial heterogeneity—not mean shifts—drives arousal-modulated hemispheric asymmetry. (**A**) Mean integration and segregation values showed no consistent hemispheric bias across individual communities or at the whole-brain (WB) level, indicating minimal imbalance in overall modulation strength. (**B**) Spatial heterogeneity, quantified as the slope of integration or segregation values ranked along a lateralization axis, was significantly steeper in every community compared with the whole-brain baseline (all p_FDR <0.0001). These effects demonstrate that hemispheric asymmetry arises from spatially patterned variation, not from uniform shifts in mean modulation. (**C**) Leave-one-out analyses revealed that this heterogeneity reflects distributed contributions from many network pairs, rather than being driven by a few extreme edges. (**D**) Contribution patterns for integration and segregation were strongly correlated (rho ≈0.72, *p*=2.35×10^–26^), indicating coordinated spatial organization across metrics. (**E–F**) Similarity in contribution patterns between communities was positively associated with similarity in communities’ intrinsic structure (integration: rho ≈ 0.79, *p*=1.35×10^–11^; segregation: rho ≈0.64, *p*=5.82×10^–7^), showing that spatial heterogeneity is constrained by each community’s underlying connectivity architecture. Statistical note: *p_FDR <0.01; **p_FDR <0.001; ***p_FDR <0.0001.

Although the mean arousal modulation on FC showed no significant lateralization, prior results suggested that its spatial pattern is highly community-specific. We, therefore, hypothesized that the key information lies in the spatial heterogeneity (distribution gradient) of the modulation strength, not the overall strength. We, therefore, quantified spatial heterogeneity by ranking network pairs along a ‘lateralization axis’ (Yang et al., 2025) and computing the slope of integration or segregation values along this axis. All communities showed slopes significantly steeper than the whole-brain baseline (all p_FDR <0.001; Figure 5B), demonstrating that lateralization arises from spatial heterogeneity—not uniform shifts.

**Figure 5—figure supplement 1.**
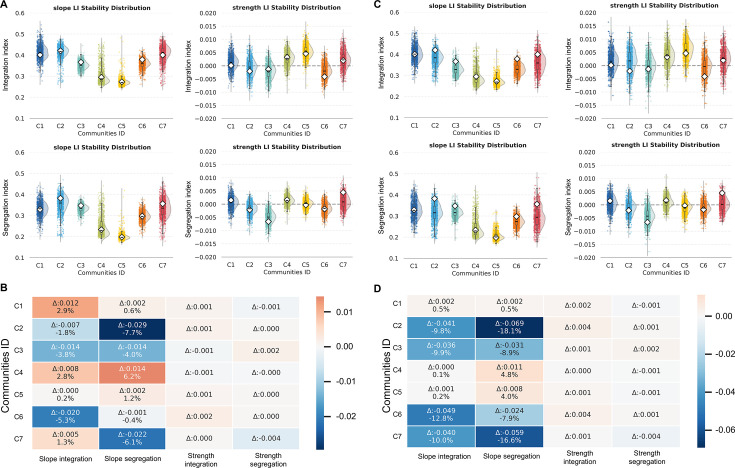
Reliability of arousal-functional connectivity (FC) coupling asymmetry across resampling strategies. (**A**) Reliability assessment using split-half resampling. Boxplots illustrate the distribution of asymmetry indices for arousal-FC coupling across 500 iterations, with diamonds indicating the original discovery findings. (**B**) The heatmap background represents the absolute deviation (Δ) between the discovery sample and the resampled mean. Numerical annotations indicate the relative error percentage (%). To prevent the statistical inflation of relative values in network pairs with minimal effects, these annotations are explicitly omitted where the $LI_{orig}$ is low (<0.05), focusing the assessment on robustly lateralized patterns. (**C–D**) Reliability assessment using participant-level resampling, following the same quantitative framework. The consistent alignment between the resampled distributions and primary findings confirms that the hemispheric asymmetry of arousal-time varying functional connectivity (tvFC) coupling is a robust biological feature rather than a result of sampling bias or specific data partitions.

Leave-one-out analyses showed that heterogeneity does not depend on a small set of extreme network pairs. Instead, most pairs contributed modestly, producing broad, community-specific gradients (Figure 5C). Contribution patterns for integration and segregation were highly correlated (rho ≈0.72, *p*=2.35×10^–26^). Furthermore, similarity in contribution patterns tracked similarity in intrinsic community structure (integration: rho ≈ 0.79, *p*=1.35×10^–11^; segregation: rho ≈ 0.64, *p*=5.82×10^–7^), indicating that spatial heterogeneity is shaped by underlying connectivity architecture (Figure 5E–F).

Thus, arousal-driven lateralization is best understood as structured spatial heterogeneity within communities, rather than gross hemispheric dominance.

The robustness of these observations was further supported by the aforementioned resampling validations (Figure 5—figure supplement 1). For the mean integration and segregation indices, the empirical values consistently fell within the center of the resampling distributions, reinforcing the absence of a uniform hemispheric mean shift across the population. Conversely, for the spatial heterogeneity metrics, the slopes observed in each community remained stable during resampling, with the empirical values largely aligned with the center of their respective distributions. This high degree of consistency confirms that the reported spatial heterogeneity is a stable and representative feature of arousal-modulated lateralization, ensuring that our findings are not skewed by specific sample compositions or outliers.

### Community structure and hemispheric asymmetry are preserved during movie watching

To assess whether these organizational principles generalize beyond the resting state, we applied the same analytic pipeline to naturalistic movie-watching data from the same participants.

**Figure 6.**
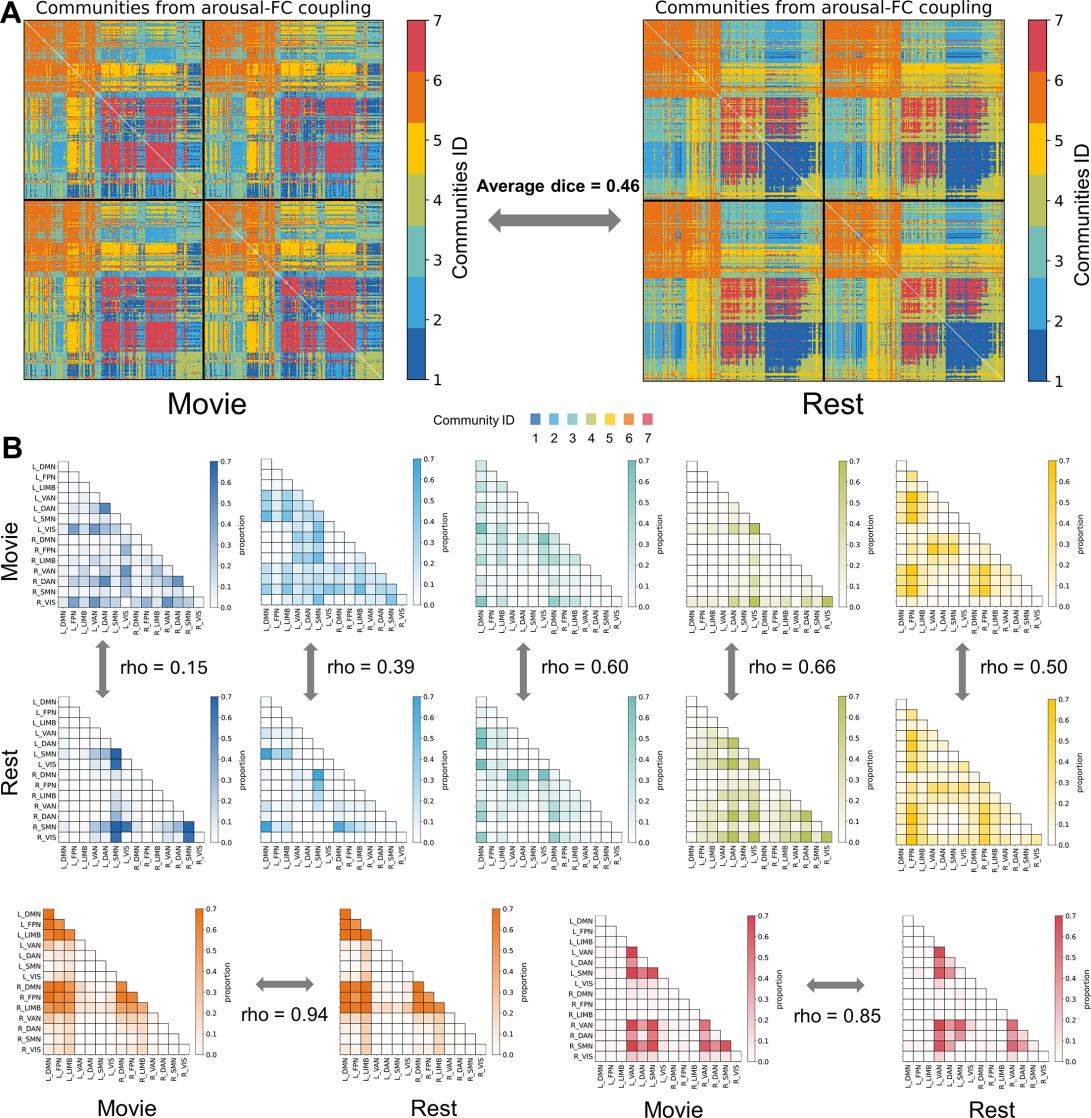
Community structure and hemispheric asymmetry of arousal–time varying functional connectivity (tvFC) coupling are preserved across resting state and movie watching. (**A**) Communities derived from rest and movie data were aligned using the Hungarian algorithm, revealing robust correspondence between paradigms (average dice ≈0.46). (**B**) Network-pair composition profiles for each community were strongly correlated across paradigms (mean rho ≈0.58), indicating that the modular organization of arousal–tvFC coupling is stable across cognitive contexts.

The seven-community structure was highly preserved across paradigms. After aligning communities using the Hungarian algorithm (Kuhn, 1955), we found that the overall community structure showed consistent correspondence (average dice ≈0.46; Figure 6A–B). One heteromodal-dominated community (community 6) showed near-perfect correspondence (rho ≈0.94), suggesting that its arousal sensitivity is largely independent of external stimulation. Other communities also demonstrated moderate cross-paradigm similarity, with only two showing low similarity—likely reflecting context-dependent modulation under rich sensory input.

These findings indicate that the modular, asymmetric organization of arousal–tvFC coupling is not specific to rest but reflects intrinsic principles that persist across cognitive contexts.

## Discussion

In this study, we investigated how moment-to-moment fluctuations in arousal shape large-scale functional connectivity in the awake human brain. By combining high-field fMRI with concurrent pupillometry, we quantified arousal–tvFC coupling at the level of individual edges and showed that arousal does not exert a uniform or diffuse influence across the connectome. Instead, it modulates connectivity through a set of seven distinct communities, each defined by characteristic network compositions and hemispheric patterns. These findings demonstrate that fluctuations in arousal, even within stable wakefulness, impose a structured and asymmetric organization on whole-brain functional interactions.

Although arousal is often conceptualized as a global modulatory state, our results show that its impact on tvFC is highly organized. Specifically, we show the presence of low-dimensional, reproducible communities suggests that arousal modulates the connectome through structured spatial patterns rather than homogeneous gain modulation. We hypothesize that this structured macroscopic architecture reflects the differentiated projection patterns of subcortical neuromodulatory systems, such as the locus coeruleus–noradrenergic pathway (Aston-Jones and Cohen, 2005; Jordan, 2024) and thalamus (Magnin et al., 2010; Lewis et al., 2015; Liu et al., 2018). This organized pattern of modulation further supports the view advocated in recent years that arousal states should be viewed as possessing spatiotemporal dynamics and regional complexity (Siclari and Tononi, 2017; Nir and de Lecea, 2023), and is strongly supported by prior work showing that spontaneous arousal fluctuations influence distributed cortical responses in selective ways (Reimer et al., 2014; McGinley et al., 2015). The hierarchical community pattern—where unimodal interactions cluster into fewer patterns while heteromodal systems participate broadly—is compatible with theories that place heteromodal cortex at the apex of large-scale integrative gradients (Mesulam, 1998; Margulies et al., 2016). Together, these observations suggest that the arousal-sensitive connectome reflects not merely regional susceptibility but an intrinsic network-level architecture, in which state-dependent modulation is aligned with established large-scale organizational principles.

A major goal of this work was to determine whether arousal imposes systematic hemispheric asymmetry on FC. We found clear evidence that it does, but in a community-specific rather than global manner. Certain communities showed rightward integration, others leftward segregation, and others no significant bias—indicating that hemispheric asymmetry emerges from the organization of arousal-sensitive connectivity motifs rather than a single overarching hemispheric dominance. This distributed asymmetry resonates with converging evidence across species: unihemispheric vigilance in birds and marine mammals (Rattenborg et al., 2000; Lyamin et al., 2016; Mascetti, 2016; Reicher et al., 2021; Fenk et al., 2023; Libourel et al., 2023), asymmetric EEG signatures related to vigilance in humans (Tamaki et al., 2016), and classic findings of right-lateralized alerting and reorienting functions (Heilman and Van Den Abell, 1980; Sturm and Willmes, 2001; Shulman et al., 2010; Corbetta and Shulman, 2011). This suggests that hemispheric specialization may arise from distinct modes of arousal-modulated reconfiguration rather than fixed structural asymmetries.

Despite the robustness of these hemispheric differences, mean integration and segregation of arousal-tvFC coupling strength across the entire community or whole brain showed minimal global lateralization. Instead, arousal-driven asymmetry manifested as spatially heterogeneous gradients within communities. Arousal does not uniformly shift connectivity toward one hemisphere; rather, it selectively amplifies lateralization in specific connectivity motifs. This distributed, gradient-like pattern complements recent work highlighting macroscale cortical gradients and manifold structure as fundamental organizational principles (Margulies et al., 2016; Huntenburg et al., 2018). We further found that communities with similar intrinsic topology exhibited similar heterogeneity signatures, suggesting that baseline connectivity architecture constrains how arousal shapes hemispheric interactions. This provides a mechanistic explanation for why traditional hemisphere-level metrics often obscure arousal-modulated asymmetries—these effects are expressed not as global biases but as structured, topology-dependent gradients.

Another important observation is the stability of arousal–tvFC organization across cognitive contexts. While the overall spatial layout of the seven-community architecture showed moderate reorganization between resting-state and naturalistic movie-watching, specific motifs—most notably communities 6 and 7—demonstrated near-perfect correspondence across paradigms. This robustness aligns with prior evidence that intrinsic connectivity organization persists across tasks (Cole et al., 2014; Krienen et al., 2014) and that spontaneous fluctuations in arousal modulate cortical dynamics even during rich sensory stimulation (Tanner et al., 2023). By demonstrating that arousal-driven network modulation generalizes across both internally and externally oriented states, our findings indicate that arousal acts as a stable organizing axis of large-scale brain communication, rather than merely a background physiological fluctuation.

Despite the important findings of this study, several limitations should be noted. First, to ensure a mathematically rigorous assessment of hemispheric asymmetry, our analysis was restricted to a symmetric cortical parcellation. Consequently, while we demonstrate that arousal-modulated connectivity follows a structured macroscopic architecture, we did not explicitly analyze the subcortical nuclei hypothesized to drive these patterns. We hypothesize that the presence of these low-dimensional cortical communities reflects coordinated motifs rather than a homogeneous gain modulation, potentially mirroring the differentiated projection patterns of subcortical neuromodulatory systems. For instance, the locus coeruleus–noradrenergic pathway (Chandler et al., 2014; Schwarz and Luo, 2015) and thalamus (Hwang et al., 2017; Shine, 2019; Müller et al., 2020; Shine et al., 2023) possess extensive yet non-uniform projections that may anchor the community-specific and hemispherically asymmetric patterns observed here. In the absence of direct subcortical-cortical integration in our current framework, this link remains hypothetical. Future investigations incorporating high-resolution subcortical data will be essential to empirically bridge the gap between these large-scale cortical communities and the underlying physiological scaffolding of the Ascending Reticular Activating System (ARAS). Second, while pupillometry is a well-established and accessible proxy for central arousal (Joshi and Gold, 2020), it remains an indirect peripheral marker. Integrating these findings with concurrent EEG will be essential to provide a more granular, multi-modal characterization of arousal states. Third, we utilized sliding-window FC to track time-varying connectivity. Although this is a robust and widely validated technique, employing alternative dynamic approaches with higher temporal resolution—such as edge FC (Faskowitz et al., 2020), multiplication of temporal derivatives (Shine et al., 2015) or dynamic conditional correlation (Lindquist et al., 2014)—may offer complementary perspectives on the transient dynamics of arousal-modulated states. Fourth, the generalizability of our approach to external cohorts warrants caution regarding pupillary data integrity. In contexts where high-fidelity eye-tracking is technically demanding—such as in clinical settings involving patients with restricted compliance or in naturalistic fMRI studies—the prevalence of blink artifacts and signal dropouts may bias the estimation of arousal-modulated states. Excessive reliance on data interpolation in such cases could artificially smooth temporal fluctuations, leading to an overestimation of community stability. Future applications should, therefore, prioritize high-frequency sampling and potentially incorporate multi-modal physiological features (e.g. respiratory or cardiac signals) to cross-validate arousal dynamics when pupillary data is suboptimal (Meissner et al., 2024; Bolt et al., 2025; Weijs et al., 2025). Fifth, the findings reported here were derived exclusively from ultra-high-field (7T) imaging data. The superior BOLD sensitivity of 7T fMRI was instrumental in resolving the fine-scale community architecture of arousal–tvFC coupling, which involves subtle signals that may be challenging to detect at lower field strengths. Given that 3T remains the most common parameter for neuroimaging research and clinical applications, future investigations are needed to determine the extent to which these organizational principles generalize to standard field strength data. Validating these communities in large-scale 3T datasets will be essential to establish their broader applicability across different imaging environments. Sixth, our findings were derived using a single high-resolution cortical parcellation. While the specific choice of atlas can influence fine-grained regional connectivity, it is important to note that our primary conclusions—such as hemispheric asymmetries and community-level preferences—were identified and interpreted at the macroscopic network and system level. By aggregating signals across broad functional systems, this approach likely mitigates the dependency on precise regional boundary definitions. Nevertheless, future studies employing alternative parcellation schemes would be valuable to further confirm that these organizational principles are not specific to the current atlas but represent a generalizable feature of the arousal-modulated connectome.

In summary, moment-to-moment fluctuations in arousal modulate functional connectivity through a small number of structured connectivity communities, each with distinct hemispheric characteristics. These asymmetries arise not from global shifts but from spatially heterogeneous gradients embedded within community structure. The reproducibility of these communities across resting state and naturalistic stimulation suggests that they reflect stable and intrinsic principles by which arousal dynamically shapes large-scale brain interactions during wakefulness.

## Materials and methods

### Participants and datasets

We used data from the Human Connectome Project (HCP) 7T dataset, which includes resting-state fMRI and naturalistic movie-watching runs with simultaneous eye tracking (Van Essen et al., 2013). Across participants, up to four resting runs and four movie runs were available, each approximately 15–16 min in length. Participant recruitment and data collection were led by Washington University and the University of Minnesota. The Institutional Review Board (IRB) at Washington University approved all experimental procedures (IRB number 201204036), and all participants provided written informed consent before data collection (Van Essen et al., 2013).

### MRI acquisition and preprocessing

All imaging was collected on a Siemens 7T scanner (TR = 1 s, TE = 22.2 ms, voxel size = 1.6 mm isotropic, multiband factor = 5900 volumes/run). We used the HCP minimally preprocessed data (Glasser et al., 2013), followed by additional denoising: linear detrending; regression of 24 head-motion parameters; regression of white matter and CSF signals; band-pass filtering (0.01–0.1 Hz); scrubbing of frames with FD >0.2 mm; and interpolation across removed frames.

### ROI parcellation

Analyses were performed using a 400-ROI symmetric parcellation with explicitly matched left–right homologues (Yan et al., 2023). Vertexwise BOLD signals were averaged within each ROI to obtain regional time series. All subsequent hemispheric analyses relied on this explicit homotopic structure.

### Eye tracking preprocessing

Pupil diameter was extracted from the raw eye-tracking stream and cleaned following established procedures (Gonzalez-Castillo et al., 2022): Removal of samples outside MRI acquisition; detection of blinks and short missing segments (<1 s); linear interpolation across missing segments; removal of brief physiologically implausible excursions (<1 ms within long closures); smoothing with a 200 ms Hanning window; down-sampling to 1 Hz to match the temporal resolution of the fMRI data. The resulting time series served as a continuous arousal index for each run.

### Quality control

The final analyzed sample for the resting-state consisted of N=139 healthy participants (mean age=29.1±3.5 years, 77 female). Runs were excluded if (a) more than 20% of frames exceeded motion thresholds, (b) eye tracking did not cover the full fMRI time series, or (c) more than 90% of samples were classified as eye closure. After applying these criteria, 485 of the initial 723 scans were retained for analysis. The same quality-control pipeline was applied to the movie-watching dataset, yielding 513 usable scans out of the original 725. After rigorous quality control, 139 participants (485 runs) were retained for the final analysis. Detailed information on data retention and run distribution per participant is summarized in Appendix 6—figure 1.

### Time-varying functional connectivity

Time-varying FC between each pair of ROIs was estimated using sliding-window correlations: window length: 30 s; step size: 5 s. Within each window, Pearson correlation coefficients were computed and Fisher-z transformed. This procedure yielded a tvFC time series for each edge in each run.

### Arousal estimation from pupil size

Pupil data were processed using the same sliding-window parameters. The mean pupil size within each window was taken as an index of moment-to-moment arousal level.

### Estimating arousal–tvFC coupling

For each functional connection, arousal–tvFC coupling was defined as the Pearson correlation between its time-varying FC and the pupil-derived arousal fluctuations across windows. Thus, each run produced a 400×400 symmetric matrix of coupling values, later vectorized into edgewise features.

These matrices were concatenated across runs to form the dataset used for community detection and all subsequent analyses.

### Community detection on arousal–tvFC coupling

To identify brain regions sharing similar arousal-related modulation profiles, we performed community detection on the edgewise coupling values. Prior to clustering, coupling values were z-scored across runs to ensure comparability. In this analytical framework, brain edges were treated as observations, while the individual runs served as feature dimensions, effectively representing each edge by its unique across-run coupling motif.

We employed the k-means clustering algorithm (Euclidean distance) to explore a range of cluster solutions from K=2–15. To ensure the stability of the results and avoid local optima, each K was repeated 250 times with random initializations. The optimal number of clusters was determined by evaluating clustering quality and reproducibility (e.g. maximizing silhouette stability). It is important to clarify that ‘communities’ in this context refer to clusters of edges that exhibit similar arousal-modulation motifs within a high-dimensional feature space, rather than topological modules typically derived from graph-theoretic algorithms like modularity maximization (Blondel et al., 2008). This procedure consistently identified seven distinct communities, each representing an arousal-sensitive connectivity motif.

### Mapping communities to networks and computing entropy

Each edge was assigned to one of seven communities. Edges were then mapped to Yeo’s 7 canonical networks (DMN, FPN, LIMB, VAN, DAN, SMN, and VIS) (Thomas Yeo et al., 2011).

For each network pair $\left (i,j\right)$, we computed the proportion $p_{i,j,k}$ of its edges belonging to each community *k*. Shannon entropy $H_{i,j}$ quantified how broadly a network pair participated across communities, calculated as:

$$\displaystyle  H_{i,j}=- \sum _{k=1}^{7}p_{i,j,k}{\rm log}_{2}\left (p_{i,j,k}\right)$$

A higher $H_{i,j}$ indicates a broader, more uniform participation across the seven communities, while a lower $H_{i,j}$ indicates that the edges are primarily concentrated in a few communities.

Additionally, the dominant community for each network pair is defined as

$$\displaystyle \mathrm{Dom}\left (i,j\right)={\rm arg}\,\underset{k}{\rm max}p_{i,j,k}$$

Entropy and dominant-community assignments jointly characterized both the diversity and primary affiliation of each network pair within the community structure.

Nodal-level community affiliation entropy $A_{r}$ quantified how broadly each region $r$ participated across communities. The entropy was calculated using the proportion $q_{r,k}$ of all incident edges of the region $r$ belonging to the community $k$:

$$\displaystyle  A_{r}=- \sum _{k=1}^{7}q_{r,k}{\rm log}_{2}\left (q_{r,k}\right)$$

### Quantifying hemispheric asymmetry: integration and segregation indices

To evaluate hemispheric biases in arousal–tvFC coupling, we categorized all functional edges into four types based on their nodal locations: LL (within left hemisphere), RR (within right hemisphere), LR (left-to-right), and RL (right-to-left). Following established frameworks for lateralization (Gotts et al., 2013), we calculated two complementary indices to capture the nature of this asymmetry.

The integration index provides a measure of the overall hemispheric dominance of arousal-modulated connections. A positive value indicates that arousal-modulated edges are preferentially concentrated in the left hemisphere (encompassing both its intra-hemispheric and commissural connections) relative to the right. It is defined as:

$$\displaystyle Integartion=\left (LL+LR\right)- \left (RR+RL\right)$$

The segregation index assesses whether arousal preferentially modulates local, intra-hemispheric communication versus long-range, inter-hemispheric communication. A positive value reflects a ‘segregated’ left-hemisphere bias, where arousal strengthens connections within the left hemisphere more than it strengthens its communication with the contralateral hemisphere. It is defined as:

$$\displaystyle Segregation=\left (LL- LR\right)- \left (RR- RL\right)$$

Indices were computed for each network pair, each community (weighted by edge count), and all significantly lateralized subsets. Statistical significance was assessed relative to a null model (see below).

### Spatial heterogeneity of lateralization

To quantify the spatial distribution characteristics of the arousal-tvFC coupling strength features (e.g. integration, segregation) within each community $k$, we first projected the edgewise coupling matrix for each participant onto the network-pair level, following the same procedure described in the previous section. The average coupling value of each network pair $\left (i,j\right)$ within each community $k$ is $C_{i,j,k}$. Network pairs were then sorted by their lateralization values to define the ‘lateralization axis’ (Yang et al., 2025).

Spatial heterogeneity was quantified by performing a linear regression of the network-pair coupling values against their rank along the $axis$:

$$\displaystyle \overline{C}_{i,j,k}=\beta _{k}\cdot {\rm axis}+\varepsilon _{i,j,k},$$

the regression slope $\beta _{k}$ indexed the spatial heterogeneity, where a larger $|\beta _{k}|$ indicates a greater difference in lateralization value within the community.

The influence of each individual network pair $\left (i,j\right)$ on the overall spatial heterogeneity $\beta _{k}$ was assessed using a leave-one-out method. The contribution $D_{i,j,k}$ was defined as the difference between the new slope $\bar{\beta }_{i,j,k}$ (after removing the pair) and the original slope $\beta _{k}$:

$$\displaystyle D_{i,j,k}=\bar{\beta }_{\left (i,j,k\right)}- \beta ^{\left (k\right)}$$

The sign of $D_{i,j,k}$ indicates its modulatory role: positive values enhance spatial heterogeneity, whereas negative values reduce it.

### Null model for hemispheric asymmetry

To determine whether observed lateralization exceeded chance, we construct a null model for hemispheric asymmetry.

For each run, ROI indices were permuted identically for rows and columns, preserving matrix symmetry and degree distribution while disrupting hemispheric structure. 10,000 permutations were performed. For each iteration, clustering and asymmetry indices were recomputed. P-values were FDR-corrected across comparisons.

### Cross-paradigm validation using movie watching

To assess the context-independence of arousal–tvFC organization, we applied all analyses to movie-watching runs. Community structures from rest and movie data were matched using Hungarian assignment (Kuhn, 1955). The overall similarity between the community architectures derived from the two paradigms was quantified by the mean Dice coefficient. Community-level correspondence was quantified by Spearman correlation between network-pair community profiles across paradigms.

### Robustness and validation

To ensure the stability and generalizability of our findings, we performed extensive robustness analyses across multiple biological scales. We first employed two complementary resampling strategies—500-iteration split-half reliability tests and participant-level resampling—with the entire analytical pipeline re-executed for each iteration. The stability of community partitions was quantified using Dice coefficients, while the reliability of hemispheric asymmetry indices was assessed by their average deviation from the full-dataset estimates. Crucially, we further confirmed that these organizational patterns were not driven by non-neural confounds, as the identified community architecture and lateralization remained highly stable even after explicitly controlling for head motion and the global signal in the arousal–tvFC coupling model. A series of sensitivity analyses regarding sliding-window parameters, temporal lags, and alternative pupillometry preprocessing pipelines further supported the robustness of our results. Detailed procedures and supporting results for these validations are provided in Appendix 1-6 and Appendix 1-6-figure1.

### Code availability

All analyses were implemented in Python (NumPy, SciPy, scikit-learn) using custom scripts. Visualization was performed with Matplotlib, Seaborn, and Surfplot. Computer codes used to calculate the communities, analyse results, and reproduce the figures of the study are openly available at https://github.com/kongxy6478/Arousal-modulates-functional-connectivity (copy archived at Kong, 2026).

## Figures and Tables

**Appendix 2—figure 1.**
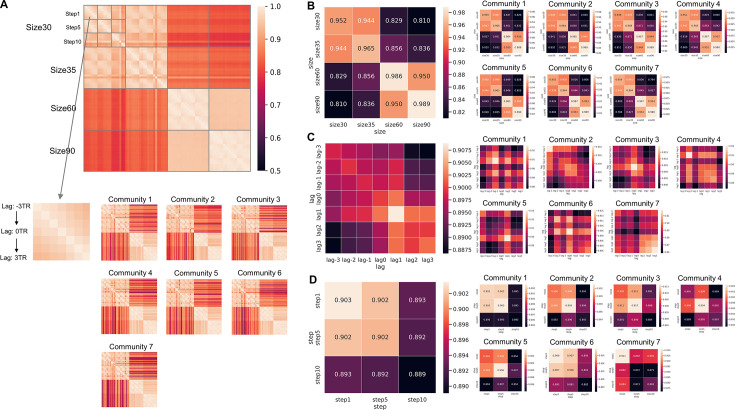
Robustness of community detection across sliding-window parameters and temporal lags. (**A**) Dice coefficients between the community topologies derived from different combinations of window size, window step, and temporal lag. The top heatmap illustrates the Dice coefficients for the overall community partition, while the bottom heatmaps show the coefficients calculated independently for each individual community. (**B–D**) Marginal consistency matrices for specific parameters. These panels display the Dice coefficients averaged across all other parameters while retaining only (**B**) window size, (**C**) temporal lag, and (**D**) window step.

**Appendix 3—figure 1.**
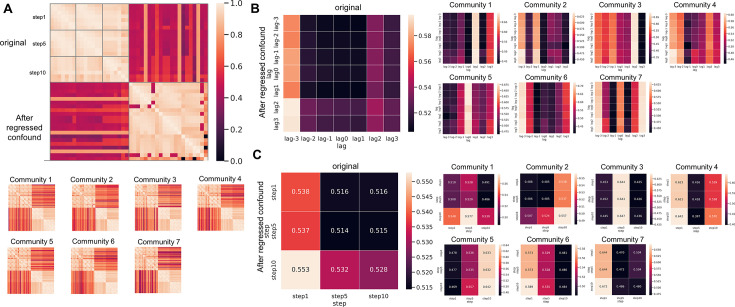
Robustness of community architecture after controlling for motion and global signal artifacts. (**A**) Dice coefficients between the original community templates and those derived after regressing out nuisance covariates (Framewise Displacement and Global Signal) across various window steps and temporal lags. The top heatmap shows the Dice coefficients for the overall community partition, while the bottom heatmaps display the coefficients for each individual community. (**B**) Marginal Dice coefficient matrix averaged across all parameters except for the temporal lag. (**C**) Marginal Dice coefficient matrix averaged across all parameters except for the window step.

**Appendix 4—figure 1.**
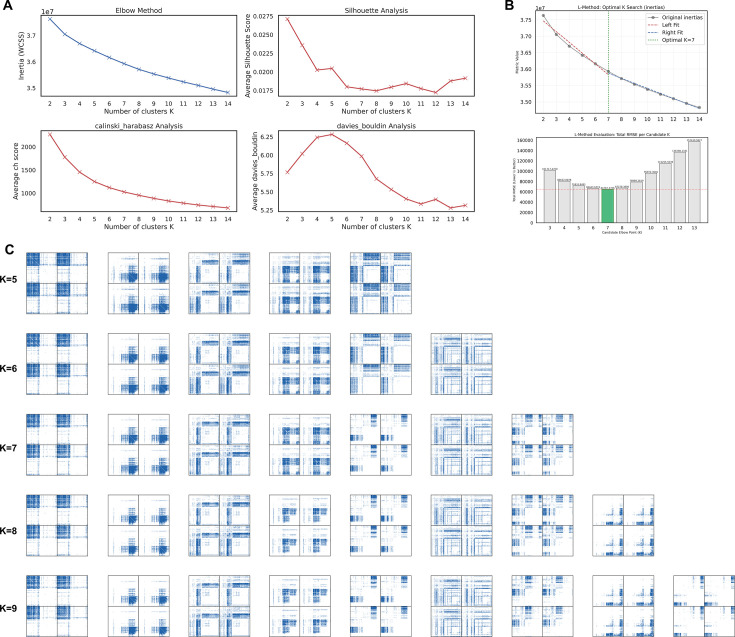
Evaluation and selection of the optimal number of communities. (**A**) Evaluation curves for four complementary clustering quality metrics across K=2–15: Within-Cluster Sum of Squares (WCSS), Davies-Bouldin Index, Calinski-Harabasz Score, and Silhouette Coefficient. (**B**) Objective determination of the ‘knee’ point using the L-method. The plot shows the total root mean squared error (RMSE) of the two-line linear regression fit as a function of K, with the minimum RMSE achieved at K=7 (indicated by the arrow). (**C**) Spatial distribution of community labels across k=5–9. The visualization demonstrates the hierarchical evolution and spatial stability of the community topologies as the number of clusters increases.

**Appendix 5—figure 1.**
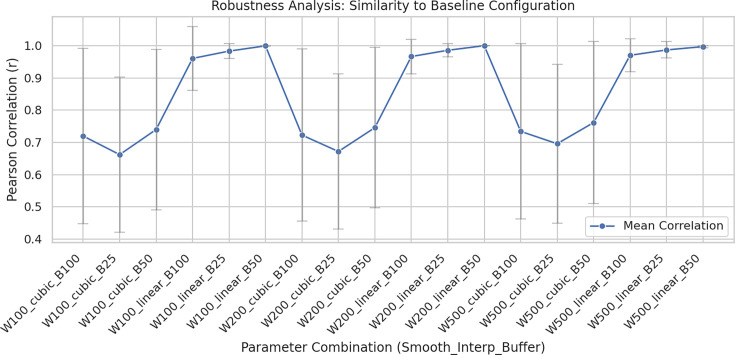
Robustness of pupil diameter time courses across different preprocessing pipelines. Line plots illustrate the Pearson correlation coefficients between the pupil diameter time courses derived from the original pipeline and those from 18 alternative preprocessing combinations. These pipelines varied across three parameters: (i) smoothing window size (100 ms, 200 ms, and 500 ms); (ii) interpolation method (linear or cubic spline); and (iii) blink buffer duration (25 ms, 50 ms, and 100 ms). Each data point represents the mean correlation across all fMRI runs, with error bars indicating ±1 standard deviation (SD).

**Appendix 6—figure 1.**
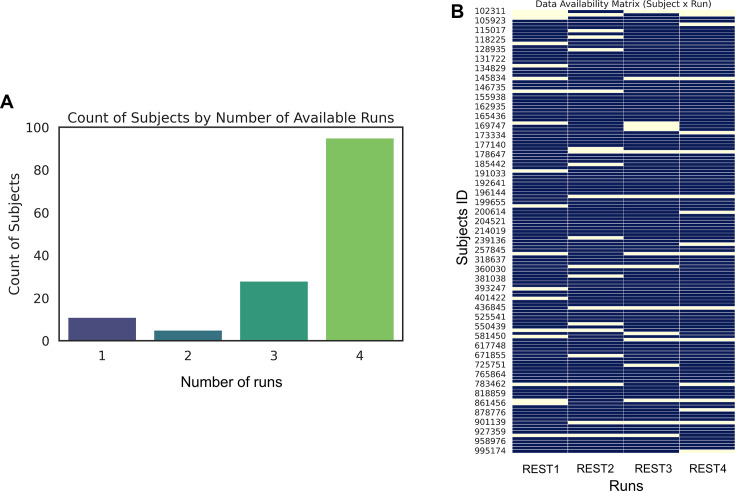
Participant-level data retention and run distribution after quality control. (**A**) Summary of valid runs per participant. The bar chart shows the number of participants who contributed 1, 2, 3, or 4 fMRI runs to the final analysis (N=139 participants). (**B**) Individual run availability matrix. The heatmap illustrates the distribution of retained runs across the four sessions (REST1 to REST4) for each participant. Each row represents an individual participant, and columns represent the four scanning sessions. Colored cells indicate that the specific run passed all quality control (QC) criteria, while white cells indicate excluded or unavailable runs.

## Data Availability

This study used publicly available data from HCP (https://www.humanconnectome.org/). The processed data and analysis code that support the findings of this study are openly available at https://github.com/kongxy6478/Arousal-modulates-functional-connectivity (copy archived at Kong, 2026).

## Additional files

MDAR checklist

## Appendix 1

### Robustness analysis of split-half reliability and participant-level resampling

To ensure that the identified community structures and hemispheric asymmetries were not driven by specific sample compositions or outliers, we performed extensive robustness validations using two complementary resampling strategies: split-half reliability and participant-level resampling. First, we conducted a split-half reliability test with 500 iterations. In each iteration, the total dataset (n=485 runs) was randomly partitioned into two independent subsets, and the entire analytical pipeline—including community detection and asymmetry quantification—was independently re-executed for each subset. The spatial consistency of community labels across iterations was quantified using the Hungarian algorithm for label alignment and evaluated via Dice coefficients (Figure 2—figure supplement 1). Second, to account for the potential dependency of multiple sessions within the same participant, we performed a participant-level resampling analysis. We constructed a sub-dataset by randomly selecting only one session per participant (N=139 participants) over 500 iterations.

The stability of asymmetry results was evaluated across multiple biological scales and analytical dimensions to capture the full landscape of the arousal-modulated connectome. This multi-level assessment encompassed: (i) network-pair hemispheric asymmetry (Figure 3—figure supplement 1), (ii) regional affiliation patterns (Figure 4—figure supplement 1), and (iii) community-averaged arousal-tvFC coupling (Figure 5—figure supplement 1). For each metric, we quantified stability by calculating the absolute deviation (Δ) between the discovery sample and the resampled mean, alongside the relative error percentage. Crucially, when evaluating relative changes, we implemented a thresholding strategy to prevent the statistical inflation of relative error in regions with low baseline effects. In regions or network pairs where the original hemispheric bias was near zero (<0.05), even trivial absolute fluctuations could produce misleadingly large relative errors. By explicitly omitting these inflated numerical annotations, we focused our robustness assessment on functionally relevant and robustly lateralized patterns. This granular approach demonstrates that the reported network-pair specific asymmetries and regional biases are consistent across diverse data partitions, reflecting an intrinsic and stable neurobiological principle of the human brain.

## Appendix 2

### Sensitivity analysis of sliding-window parameters and temporal lags

To evaluate the robustness of the identified community structures and their hemispheric asymmetries, we performed extensive sensitivity analyses across varying methodological parameters.

First, we assessed the dependence of our results on sliding-window configurations. The analytical pipeline was repeated using a range of window lengths (30 s, 35 s, 60 s, and 90 s) and sliding steps (1 s, 5 s, and 10 s). The spatial similarity between the resulting community topologies and the original configuration (window length = 30 s, step = 5 s) was quantified using Dice coefficients. As shown in Appendix 2—figure 1, the Dice coefficients for both the overall community structure and individual communities remained consistently above 0.8 across various window parameters (Appendix 2—figure 1A, B and D). These findings demonstrate that the observed arousal-modulated connectivity organization is not dependent on specific sliding-window settings.

Second, considering the hemodynamic response function (HRF) delay and the complex temporal coupling between pupil fluctuations and neural activity, we conducted a lagged cross-correlation analysis. Following established literature on the temporal relationship between tvFC and pupil dynamics, we introduced temporal lags ranging from –3 TR to +3 TR (–3s to 3s) to the pupil time course. We then re-estimated the community structures and assessed their consistency using Dice coefficients. The results (Appendix 2—figure 1C) show that the core community partitions, and lateralization patterns remain stable within physiologically plausible temporal lags, confirming that our primary findings are not biased by the zero-lag assumption.

## Appendix 3

### Robustness analysis of controlling for nuisance covariates

To ensure that the identified community structures and their hemispheric asymmetries were not confounded by head motion or non-specific physiological noise, we performed an additional robustness analysis by including nuisance covariates. This is particularly important as pupil diameter, our primary arousal index, is known to covary with head motion, blinks, and non-specific physiological noise, which could potentially bias the estimation of arousal-tvFC coupling.

Specifically, when calculating the arousal-tvFC coupling, we employed partial correlation to regress out the following variables: (i) head motion parameters (quantified by Framewise Displacement, FD) and (ii) the global signal (defined as the mean signal across all gray matter voxels). We then re-executed the community detection using these controlled coupling maps.

As shown in Appendix 3—figure 1, the resulting community topologies remained highly consistent with our primary findings, with Dice coefficients remaining stable across various lag settings even after accounting for these noise components. It is important to note that we did not regress out the eye close ratio, as cumulative evidence suggests that eyelid closure is an intrinsic and valid indicator of arousal levels; controlling for it would likely diminish the biologically relevant coupling between arousal fluctuations and functional connectivity (Sommer and Golz, 2010; Chang et al., 2016; Gonzalez-Castillo et al., 2022). These results confirm that the reported arousal-modulated connectivity organization reflects genuine neural dynamics rather than motion or global artifacts.

## Appendix 4

### Control analysis on the optimal cluster number

To ensure that the identified connectivity communities reflect a stable and intrinsic organizational feature of the brain rather than sensitivity to specific methodological decisions, we expanded our evaluation of the clustering solutions using multiple complementary criteria.

As shown in Appendix 4—figure 1, we evaluated the clustering performance for K ranging from 2 to 15 using four distinct metrics: (i) Within-Cluster Sum of Squares (WCSS), reflecting cluster compactness; (ii) Davies-Bouldin Index (DBI), assessing the ratio of within-cluster distances to between-cluster distances; (iii) Calinski-Harabasz (CH) Score, measuring the ratio of between-cluster dispersion to within-cluster dispersion; and (iv) Silhouette Coefficient, evaluating how similar an object is to its own cluster compared to other clusters (Appendix 4—figure 1A).

To objectively identify the ‘elbow’ point where increasing K yields diminishing returns, we applied the L-method (Salvador and Chan, 2004) to the evaluation curves. Specifically, we minimized the total root mean squared error (RMSE) of a two-line linear regression fit across the range of K. The RMSE reached its minimum at K=7 (Appendix 4—figure 1B), represents the optimal balance between model complexity and data fit. Furthermore, we visualized the community topologies across different K values (Appendix 4—figure 1C) to demonstrate that the core spatial organizational principles (e.g. the separation of unimodal and transmodal connectivity) remain consistent across a reasonable range of K. These multi-dimensional evaluations collectively confirm that seven communities provide a parsimonious and stable representation of the arousal-modulated connectome.

## Appendix 5

### Robustness of pupil diameter time courses across different preprocessing pipelines

Given that pupil diameter serves as the primary index of arousal in this study, it is critical to ensure that the estimated arousal fluctuations are not idiosyncratic to specific preprocessing choices. We systematically evaluated the robustness of the pupil diameter time courses by comparing our original pipeline with 18 alternative preprocessing combinations.

These pipelines involved variations in three key parameters: (i) temporal smoothing (window sizes of 100ms, 200ms, and 500 ms), (ii) artifact interpolation (linear vs. cubic spline), and (iii) blink buffering (durations of 25 ms, 50 ms, and 100 ms). For each fMRI run, we calculated the Pearson correlation coefficient between the pupil time courses derived from these alternative pipelines and the original one.

As illustrated in Appendix 5—figure 1, the mean correlation coefficients across all runs remained exceptionally high (all *r*>0.65) regardless of the parameter combinations. The minimal variance (indicated by SD) further confirms the stability of the pupil signal across different sessions and participants. These results demonstrate that the extracted arousal dynamics are highly robust to reasonable changes in pupillometry preprocessing rules, ensuring the reliability of the subsequent arousal-tvFC coupling analyses.

## Appendix 6

### Data quality control and sample retention

To ensure the reliability of the arousal-connectivity analysis, we implemented a rigorous quality control (QC) pipeline for the fMRI data. The participant-level data retention and the distribution of valid runs are summarized in Appendix 6—figure 1. Out of the initial dataset, a total of 139 participants (contributing 485 runs) met the inclusion criteria, which required at least one high-quality resting-state session per subject.

The distribution of available data was characterized across two dimensions: (i) run-wise consistency per participant, showing the number of participants who contributed one to four valid runs (Appendix 6—figure 1A), and (ii) session-specific availability, mapping the retention of specific runs across the four sessions (REST1 to REST4) for each individual subject (Appendix 6—figure 1B). This detailed mapping ensures that the longitudinal sampling density was sufficient to capture stable individual arousal-connectivity coupling while accounting for potential data loss due to excessive head motion or physiological artifacts.

## References

[bib1] Aston-Jones G, Cohen JD (2005). An integrative theory of locus coeruleus-norepinephrine function: adaptive gain and optimal performance. Annual Review of Neuroscience.

[bib2] Banks MI, Krause BM, Endemann CM, Campbell DI, Kovach CK, Dyken ME, Kawasaki H, Nourski KV (2020). Cortical functional connectivity indexes arousal state during sleep and anesthesia. NeuroImage.

[bib3] Blondel VD, Guillaume JL, Lambiotte R, Lefebvre E (2008). Fast unfolding of communities in large networks. Journal of Statistical Mechanics.

[bib4] Bolt T, Wang S, Nomi JS, Setton R, Gold BP, deB Frederick B, Yeo BTT, Chen JJ, Picchioni D, Duyn JH, Spreng RN, Keilholz SD, Uddin LQ, Chang C (2025). Autonomic physiological coupling of the global fMRI signal. Nature Neuroscience.

[bib5] Breeden AL, Siegle GJ, Norr ME, Gordon EM, Vaidya CJ (2017). Coupling between spontaneous pupillary fluctuations and brain activity relates to inattentiveness. The European Journal of Neuroscience.

[bib6] Chandler DJ, Gao WJ, Waterhouse BD (2014). Heterogeneous organization of the locus coeruleus projections to prefrontal and motor cortices. PNAS.

[bib7] Chang C, Leopold DA, Schölvinck ML, Mandelkow H, Picchioni D, Liu X, Ye FQ, Turchi JN, Duyn JH (2016). Tracking brain arousal fluctuations with fMRI. PNAS.

[bib8] Chow HM, Horovitz SG, Carr WS, Picchioni D, Coddington N, Fukunaga M, Xu Y, Balkin TJ, Duyn JH, Braun AR (2013). Rhythmic alternating patterns of brain activity distinguish rapid eye movement sleep from other states of consciousness. PNAS.

[bib9] Cole MW, Bassett DS, Power JD, Braver TS, Petersen SE (2014). Intrinsic and task-evoked network architectures of the human brain. Neuron.

[bib10] Corbetta M, Shulman GL (2011). Spatial neglect and attention networks. Annual Review of Neuroscience.

[bib11] Damaraju E, Tagliazucchi E, Laufs H, Calhoun VD (2020). Connectivity dynamics from wakefulness to sleep. NeuroImage.

[bib12] Demertzi A, Tagliazucchi E, Dehaene S, Deco G, Barttfeld P, Raimondo F, Martial C, Fernández-Espejo D, Rohaut B, Voss HU, Schiff ND, Owen AM, Laureys S, Naccache L, Sitt JD (2019). Human consciousness is supported by dynamic complex patterns of brain signal coordination. Science Advances.

[bib13] Faskowitz J, Esfahlani FZ, Jo Y, Sporns O, Betzel RF (2020). Edge-centric functional network representations of human cerebral cortex reveal overlapping system-level architecture. Nature Neuroscience.

[bib14] Fenk LA, Riquelme JL, Laurent G (2023). Interhemispheric competition during sleep. Nature.

[bib15] Glasser MF, Sotiropoulos SN, Wilson JA, Coalson TS, Fischl B, Andersson JL, Xu J, Jbabdi S, Webster M, Polimeni JR, Van Essen DC, Jenkinson M, WU-Minn HCP Consortium (2013). The minimal preprocessing pipelines for the Human Connectome Project. NeuroImage.

[bib16] Gonzalez-Castillo J, Fernandez IS, Handwerker DA, Bandettini PA (2022). Ultra-slow fMRI fluctuations in the fourth ventricle as a marker of drowsiness. NeuroImage.

[bib17] Gotts SJ, Jo HJ, Wallace GL, Saad ZS, Cox RW, Martin A (2013). Two distinct forms of functional lateralization in the human brain. PNAS.

[bib18] Heilman KM, Van Den Abell T (1980). Right hemisphere dominance for attention: the mechanism underlying hemispheric asymmetries of inattention (neglect). Neurology.

[bib19] Huang Z, Zhang J, Wu J, Mashour GA, Hudetz AG (2020). Temporal circuit of macroscale dynamic brain activity supports human consciousness. Science Advances.

[bib20] Huntenburg JM, Bazin PL, Margulies DS (2018). Large-scale gradients in human cortical organization. Trends in Cognitive Sciences.

[bib21] Hwang K, Bertolero MA, Liu WB, D’Esposito M (2017). The human thalamus is an integrative hub for functional brain networks. The Journal of Neuroscience.

[bib22] Jang H, Mashour GA, Hudetz AG, Huang Z (2024). Measuring the dynamic balance of integration and segregation underlying consciousness, anesthesia, and sleep in humans. Nature Communications.

[bib23] Jordan R (2024). The locus coeruleus as a global model failure system. Trends in Neurosciences.

[bib24] Joshi S, Gold JI (2020). Pupil size as a window on neural substrates of cognition. Trends in Cognitive Sciences.

[bib25] Kong X (2026). https://archive.softwareheritage.org/swh:1:dir:f7e3a4a3155c07074921f108debc1d33ad83d09a;origin=https://github.com/kongxy6478/Arousal-modulates-functional-connectivity;visit=swh:1:snp:3683c2f6802a16297bd23bd7c7222d337f63591e;anchor=swh:1:rev:86261f04b572686dd780ddf07e8921408cd2f7b8.

[bib26] Krienen FM, Yeo BTT, Buckner RL (2014). Reconfigurable task-dependent functional coupling modes cluster around a core functional architecture. Philosophical Transactions of the Royal Society B.

[bib27] Kuhn HW (1955). The Hungarian method for the assignment problem. Naval Research Logistics Quarterly.

[bib28] Lewis LD, Voigts J, Flores FJ, Schmitt LI, Wilson MA, Halassa MM, Brown EN (2015). Thalamic reticular nucleus induces fast and local modulation of arousal state. eLife.

[bib29] Libourel PA, Lee WY, Achin I, Chung H, Kim J, Massot B, Rattenborg NC (2023). Nesting chinstrap penguins accrue large quantities of sleep through seconds-long microsleeps. Science.

[bib30] Lindquist MA, Xu Y, Nebel MB, Caffo BS (2014). Evaluating dynamic bivariate correlations in resting-state fMRI: A comparison study and a new approach. NeuroImage.

[bib31] Liu X, de Zwart JA, Schölvinck ML, Chang C, Ye FQ, Leopold DA, Duyn JH (2018). Subcortical evidence for a contribution of arousal to fMRI studies of brain activity. Nature Communications.

[bib32] Lloyd B, de Voogd LD, Mäki-Marttunen V, Nieuwenhuis S (2023). Pupil size reflects activation of subcortical ascending arousal system nuclei during rest. eLife.

[bib33] Lyamin OI, Lapierre JL, Kosenko PO, Kodama T, Bhagwandin A, Korneva SM, Peever JH, Mukhametov LM, Siegel JM (2016). Monoamine release during unihemispheric sleep and unihemispheric waking in the fur seal. Sleep.

[bib34] Magnin M, Rey M, Bastuji H, Guillemant P, Mauguière F, Garcia-Larrea L (2010). Thalamic deactivation at sleep onset precedes that of the cerebral cortex in humans. PNAS.

[bib35] Margulies DS, Ghosh SS, Goulas A, Falkiewicz M, Huntenburg JM, Langs G, Bezgin G, Eickhoff SB, Castellanos FX, Petrides M, Jefferies E, Smallwood J (2016). Situating the default-mode network along a principal gradient of macroscale cortical organization. PNAS.

[bib36] Mascetti GG (2016). Unihemispheric sleep and asymmetrical sleep: behavioral, neurophysiological, and functional perspectives. Nature and Science of Sleep.

[bib37] McGinley MJ, Vinck M, Reimer J, Batista-Brito R, Zagha E, Cadwell CR, Tolias AS, Cardin JA, McCormick DA (2015). Waking state: Rapid variations modulate neural and behavioral responses. Neuron.

[bib38] Meissner SN, Bächinger M, Kikkert S, Imhof J, Missura S, Carro Dominguez M, Wenderoth N (2024). Self-regulating arousal via pupil-based biofeedback. Nature Human Behaviour.

[bib39] Mesulam MM (1998). From sensation to cognition. Brain.

[bib40] Müller EJ, Munn B, Hearne LJ, Smith JB, Fulcher B, Arnatkevičiūtė A, Lurie DJ, Cocchi L, Shine JM (2020). Core and matrix thalamic sub-populations relate to spatio-temporal cortical connectivity gradients. NeuroImage.

[bib41] Nir Y, de Lecea L (2023). Sleep and vigilance states: Embracing spatiotemporal dynamics. Neuron.

[bib42] Podvalny E, King LE, He BJ (2021). Spectral signature and behavioral consequence of spontaneous shifts of pupil-linked arousal in human. eLife.

[bib43] Rattenborg NC, Amlaner CJ, Lima SL (2000). Behavioral, neurophysiological and evolutionary perspectives on unihemispheric sleep. Neuroscience & Biobehavioral Reviews.

[bib44] Reicher V, Kis A, Simor P, Bódizs R, Gácsi M (2021). Interhemispheric asymmetry during NREM sleep in the dog. Scientific Reports.

[bib45] Reimer J, Froudarakis E, Cadwell CR, Yatsenko D, Denfield GH, Tolias AS (2014). Pupil fluctuations track fast switching of cortical states during quiet wakefulness. Neuron.

[bib46] Salvador S, Chan P (2004). Determining the number of clusters/segments in hierarchical clustering/segmentation algorithms.

[bib47] Schneider M, Hathway P, Leuchs L, Sämann PG, Czisch M, Spoormaker VI (2016). Spontaneous pupil dilations during the resting state are associated with activation of the salience network. NeuroImage.

[bib48] Schwarz LA, Luo L (2015). Organization of the locus coeruleus-norepinephrine system. Current Biology.

[bib49] Shine JM, Koyejo O, Bell PT, Gorgolewski KJ, Gilat M, Poldrack RA (2015). Estimation of dynamic functional connectivity using multiplication of temporal derivatives. NeuroImage.

[bib50] Shine JM (2019). Neuromodulatory influences on integration and segregation in the brain. Trends in Cognitive Sciences.

[bib51] Shine JM, Lewis LD, Garrett DD, Hwang K (2023). The impact of the human thalamus on brain-wide information processing. Nature Reviews. Neuroscience.

[bib52] Shulman GL, Pope DLW, Astafiev SV, McAvoy MP, Snyder AZ, Corbetta M (2010). Right hemisphere dominance during spatial selective attention and target detection occurs outside the dorsal frontoparietal network. The Journal of Neuroscience.

[bib53] Siclari F, Tononi G (2017). Local aspects of sleep and wakefulness. Current Opinion in Neurobiology.

[bib54] Sobczak F, Pais-Roldán P, Takahashi K, Yu X (2021). Decoding the brain state-dependent relationship between pupil dynamics and resting state fMRI signal fluctuation. eLife.

[bib55] Sommer D, Golz M (2010). Evaluation of PERCLOS based current fatigue monitoring technologies.

[bib56] Sturm W, Willmes K (2001). On the functional neuroanatomy of intrinsic and phasic alertness. NeuroImage.

[bib57] Tagliazucchi E, Roseman L, Kaelen M, Orban C, Muthukumaraswamy SD, Murphy K, Laufs H, Leech R, McGonigle J, Crossley N, Bullmore E, Williams T, Bolstridge M, Feilding A, Nutt DJ, Carhart-Harris R (2016). Increased global functional connectivity correlates with LSD-induced ego dissolution. Current Biology.

[bib58] Tamaki M, Bang JW, Watanabe T, Sasaki Y (2016). Night watch in one brain hemisphere during sleep associated with the first-night effect in humans. Current Biology.

[bib59] Tanner JC, Faskowitz J, Byrge L, Kennedy DP, Sporns O, Betzel RF (2023). Synchronous high-amplitude co-fluctuations of functional brain networks during movie-watching. Imaging Neuroscience.

[bib60] Thomas Yeo BT, Krienen FM, Sepulcre J, Sabuncu MR, Lashkari D, Hollinshead M, Roffman JL, Smoller JW, Zöllei L, Polimeni JR, Fischl B, Liu H, Buckner RL (2011). The organization of the human cerebral cortex estimated by intrinsic functional connectivity. Journal of Neurophysiology.

[bib61] Van Essen DC, Smith SM, Barch DM, Behrens TEJ, Yacoub E, Ugurbil K, WU-Minn HCP Consortium (2013). The WU-Minn human connectome project: An overview. NeuroImage.

[bib62] Weijs ML, Missura S, Potok-Szybińska W, Bächinger M, Badii B, Carro-Domínguez M, Wenderoth N, Meissner SN (2025). Modulating cortical excitability and cortical arousal by pupil self-regulation. Nature Communications.

[bib63] Yan X, Kong R, Xue A, Yang Q, Orban C, An L, Holmes AJ, Qian X, Chen J, Zuo XN, Zhou JH, Fortier MV, Tan AP, Gluckman P, Chong YS, Meaney MJ, Bzdok D, Eickhoff SB, Yeo BTT (2023). Homotopic local-global parcellation of the human cerebral cortex from resting-state functional connectivity. NeuroImage.

[bib64] Yang H, Wu G, Li Y, Xu X, Cong J, Xu H, Ma Y, Li Y, Chen R, Pines A, Xu T, Sydnor VJ, Satterthwaite TD, Cui Z (2025). Connectional axis of individual functional variability: Patterns, structural correlates, and relevance for development and cognition. PNAS.

[bib65] Yellin D, Berkovich-Ohana A, Malach R (2015). Coupling between pupil fluctuations and resting-state fMRI uncovers a slow build-up of antagonistic responses in the human cortex. NeuroImage.

