# Supplementary material for: Arousal modulates functional connectivity through structured and hemispherically asymmetric community architecture during wakefulness
Source: eLife. 2026 Jun 24;15:RP110294. doi: 10.7554/eLife.110294 (PMC13293607; doi:10.7554/eLife.110294)
Supplement: Peer review history

# Editor report: eLife Assessment

**DOI:** 10.7554/eLife.110294.3.sa0

**Authors:** Alex Fornito

**Affiliations:** Monash University Australia

**Keywords:** Convincing, Valuable

This study offers a **valuable** analysis of how moment-to-moment fluctuations in arousal are associated with structured, non-uniform patterns of brain-wide functional connectivity during wakefulness. Using data-driven analyses of resting-state and naturalistic fMRI with eye tracking, the authors present **convincing** evidence that arousal is a dynamic, continuous process that shapes brain activity in a structured way beyond a simple global effect. This paper sheds light on the link between brain activity and ongoing fluctuations in arousal and will be of interest to researchers studying large-scale brain functional organization and links between the brain and body.

# Referee report: Reviewer #1 (Public review)

**DOI:** 10.7554/eLife.110294.3.sa1

Summary:

In this study, the authors aim to characterize how moment-to-moment fluctuations in arousal during wakefulness shape large-scale functional brain connectivity. Using pupil diameter as an index of arousal and high-field functional imaging, they seek to determine whether arousal-related modulation of connectivity is uniform across the brain or organized into structured patterns, and whether such patterns show hemispheric asymmetry. The work further aims to assess whether these organizational features generalize across resting-state and naturalistic viewing conditions.

Strengths:

The study addresses an important and timely question regarding how spontaneous variations in arousal influence whole-brain communication during wakefulness. The dataset is rich, combining high-field imaging with concurrent physiological measurements, and the analyses are ambitious in scope. A key strength is the attempt to move beyond region-based effects and to describe arousal-related modulation at the level of large-scale connectivity organization. The comparison across rest and movie viewing provides useful context and suggests a degree of consistency across behavioral states.

Weaknesses

All analyses are based on 7T ultra-high-field imaging. The manuscript does not address whether the reported arousal-related patterns, including the community structure and hemispheric asymmetries, are expected to be reproducible at standard 3T field strengths. It therefore remains unclear whether the findings depend critically on the use of high-field data or whether they would generalize to more widely available datasets, limiting the broader applicability of the results.

# Referee report: Reviewer #2 (Public review)

**DOI:** 10.7554/eLife.110294.3.sa2

Summary:

This manuscript addresses a clear and widely relevant question: how ongoing fluctuations in alertness during wakefulness relate to large scale patterns of coordinated brain activity. The authors combine high field magnetic resonance imaging with simultaneous pupil measurements, and they compute an edgewise measure of arousal-related coupling for every pair of regions. Their main contribution is to show that arousal-related coupling is low dimensional and organized into seven reproducible "connectivity communities", each with characteristic network pair compositions. A secondary contribution is the observation that these communities exhibit systematic but community-specific hemispheric asymmetries, including a striking left/right dissociation within the ventral attention network, where the left side participates broadly across communities while the right side forms a more cohesive, segregated arousal responsive module. A final contribution is cross-context generalization: the same organizational structure and lateralization signatures are largely preserved during naturalistic movie watching.

Strengths:

(1) The paper moves beyond state contrasts and quantifies arousal related modulation continuously within wakefulness, directly addressing a gap highlighted in the Introduction.

(2) The hemispheric asymmetry result is not framed as a crude global dominance effect; the authors explicitly test and argue that the key signal lies in structured spatial heterogeneity rather than mean shifts.

(3) The cross-paradigm replication in movie watching is a strong design choice and supports the claim that the organizational motifs are not limited to unconstrained rest.

(4) Arousal effects on BOLD signals and on pupil size can have different delays. The authors have now tested lagged relationships (for example shifting the pupil series forward and backward) to show that the main community structure and lateralization results are not sensitive to an arbitrary temporal alignment.

(5) Time resolved connectivity results are now shown to be robust to changes in parameters.

# Referee report: Reviewer #3 (Public review)

**DOI:** 10.7554/eLife.110294.3.sa3

Summary:

The paper investigates neural fluctuations underlying arousal using a combination of resting state/naturalistic movie watching fMRI and eye tracking data. The authors have used several data driven approaches, including time varying sliding window analyses and clustering methods, to characterize large scale brain organization and hemispheric asymmetries associated with arousal fluctuations. This is an interesting study framing arousal as a dynamic, continuously varying process rather than a discrete state. Overall, the manuscript is well written and the authors have provided sufficient details about the methodological choices, their impact on the results, along with the limitations of the study.

Strengths:

This is an interesting study framing arousal as a dynamic, continuously varying process rather than a discrete state. Overall, the manuscript is well written and provides sufficient methodological and analytical details to evaluate the results.

Weakness:

While the study provides new insights regarding neural processes underlying arousal, future studies may be needed to further examine the implications of identified cluster and patterns.

# Author comment: Author response

**DOI:** 10.7554/eLife.110294.3.sa4

**Authors:** Xiangyu Kong, Siyu Li, Gaolang Gong

**Affiliations:** State Key Laboratory of Cognitive Neuroscience and Learning and IDG/McGovern Institute for Brain Research,Beijing Normal University Beijing China; State Key Laboratory of Cognitive Neuroscience and Learning and IDG/McGovern Institute for Brain Research, Beijing Normal University Beijing China; Beijing Normal University Beijing China

The following is the authors’ response to the original reviews.

**Public Reviews:**

**Reviewer #1 (Public review):**

(1) First, a central claim is that arousal modulates functional connectivity in a hemispherically asymmetric and community-specific manner. Although structured asymmetries are demonstrated at the group level, it remains unclear whether these effects reflect a stable neurobiological principle or arise from high-dimensional, connection-wise analyses that are sensitive to sampling variability. Given the interpretive weight placed on hemispheric lateralization, stronger evidence of robustness and individual-level consistency would be necessary to support this conclusion.

We appreciate your critical comments on the robustness of our lateralization findings. We fully agree with you that it is essential to demonstrate that the observed hemispheric asymmetries reflect a stable neurobiological principle rather than an artifact of sampling variability or high-dimensional noise. To address this concern, we performed two rigorous validation analyses using 500-iteration resampling schemes, consisting of a split-half reliability test and a participant-level consistency assessment.

First, to ensure our findings do not depend on specific sample compositions, we conducted a split-half reliability test where the dataset was randomly partitioned into two independent subgroups over 500 iterations. As shown in Figure S1A, the community labels maintained high spatial consistency across iterations (as evidenced by the confusion matrix and Dice coefficient distributions), and our original findings—including network-pair community architecture (Fig. S2A), regional affiliation patterns (Fig. S3A-B), and arousal–tvFC coupling lateralization (Fig. S4A-B)—were consistently situated at the center of the iteration distributions.

Second, to account for potential within-participant dependencies in the HCP 7T dataset, we performed a participant-level resampling analysis (N = 139). By randomly selecting a different session for each participant across 500 iterations, we confirmed that the community architecture and hemispheric biases remain robust even under this strict control (Figure S1A, S2B, S3C-D and S4C-D). Collectively, these additional analyses provide strong evidence that the hemispheric lateralization we reported is not a byproduct of sampling bias, but instead represents a stable organizational principle of the arousal-modulated connectome.

(2) Second, all analyses are based on ultra-high-field imaging. The manuscript does not address whether the reported arousal-related patterns, including the community structure and hemispheric asymmetries, are expected to be reproducible at standard field strengths. It therefore remains unclear whether the findings depend critically on the use of high-field data or whether they would generalize to more widely available datasets, limiting the broader applicability of the results.

We appreciate your constructive comments on the generalizability of our findings across different field strengths.

As you noted, our primary motivation for employing 7T ultra-high-field imaging was to leverage its superior signal-to-noise ratio (SNR) and significantly enhanced BOLD sensitivity. These technical advantages were instrumental in capturing the subtle, moment-to-moment coupling between spontaneous pupillary fluctuations and tvFC—signals that might be close to the detection threshold in standard field strength environments.

However, we fully recognize your point that 3T remains the standard in most clinical and research settings. In the revised manuscript, we have added a dedicated discussion to address this (page 21, lines 447-456):

“Fifth, the findings reported here were derived exclusively from ultra-high-field (7T) imaging data. The superior BOLD sensitivity of 7T fMRI was instrumental in resolving the fine-scale community architecture of arousal–tvFC coupling, which involves subtle signals that may be challenging to detect at lower field strengths. Given that 3T remains the most common parameter for neuroimaging research and clinical applications, future investigations are needed to determine the extent to which these organizational principles generalize to standard field strength data. Validating these motifs in large-scale 3T datasets will be essential to establish their broader applicability across different imaging environments.”

(3) Third, arousal-connectivity coupling is assessed using zero-lag correlations between pupil diameter and time-resolved connectivity estimates. Physiological and hemodynamic considerations suggest that pupil-linked arousal and blood-based imaging signals may exhibit systematic temporal delays. The absence of analyses examining sensitivity to such delays raises the possibility that the reported coupling patterns depend on a specific temporal alignment assumption.

Given the inherent delay of the hemodynamic response function (HRF) and the complex temporal relationship between pupillary dynamics and neural activity, we conducted an additional lagged cross-correlation analysis to test the sensitivity of our findings. Following established frameworks for linking BOLD signals with pupillometry (Yellin et al., 2015; Gonzalez-Castillo et al., 2022; Lloyd et al., 2023), we systematically shifted the pupil time series relative to the fMRI data by -3 TR to +3 TR (-3s to +3s) and evaluated the consistency of the community architecture across these different lags using Dice coefficients.

As shown in Figure S5, these results demonstrate that the community organization remain stable across the tested range of physiological delays. This stability indicates that the arousal-modulated communities we reported are not specific to the zero-lag assumption but instead persist throughout the physiologically plausible lag window. Consequently, our findings reflect a robust neurobiological phenomenon rather than an artifact of a specific temporal alignment.

(4) Fourth, the estimation of time-resolved connectivity relies on a single choice of sliding-window length. The manuscript does not examine whether the reported patterns are stable across different window sizes. Given ongoing concerns about parameter dependence in time-resolved connectivity analyses, sensitivity analyses would be important to establish that the findings are not artifacts of a particular analytical choice.

To ensure that our findings are not artifacts of a specific analytical choice, we performed an exhaustive sensitivity analysis by repeating our entire pipeline across a wide range of window lengths (30s, 35s, 60s, and 90s) and step sizes (1s, 5s, and 10s). We then employed Dice coefficients to quantify the topological similarity between these alternative configurations and our original parameters (30s window, 5s step).

As shown in Figure S5, our results demonstrate high topological consistency, with Dice coefficients for community structures remaining consistently above 0.8 across all tested parameter combinations. These findings provide strong evidence that the arousal-modulated organizational principles we reported are inherent to the data rather than being driven by specific analytical choices in the sliding-window setup.

(5) Finally, the identification of seven connectivity communities is a central result, yet the justification for this choice relies primarily on a single clustering quality measure. In practice, evaluation of clustering solutions typically draws on multiple complementary criteria, including measures of compactness and separation, approaches for selecting the number of clusters, and assessments of stability under resampling. Without such complementary evaluations, it is difficult to determine whether the reported community structure reflects a stable organizational feature or sensitivity to specific methodological decisions.

We agree that relying on a single measure can be limiting, and in the revised manuscript, we have implemented a comprehensive multi-criteria evaluation to justify our selection of K=7. To ensure the robustness of the community partition, we expanded our analysis to include several complementary indices, such as the Davies-Bouldin Index, Calinski-Harabasz Score, and Silhouette Coefficient, alongside the original Within-Cluster Sum of Squares (WCSS), as detailed in Figure S7A.

To further minimize subjective bias in "elbow" detection, we utilized the L-method (Salvador & Chan, 2004), which identifies the optimal K by minimizing the combined root-mean-square error (RMSE) of two linear regression segments. As illustrated in Figure S7B, the RMSE was minimized at K=7, providing a robust mathematical basis for our partition. Furthermore, we systematically visualized the community maps across a range of granularities from K=5 to 9 (Figure S7C). This stability analysis demonstrates that the fundamental topological features and the resulting hemispheric asymmetries are not transient artifacts of a specific K but are consistently preserved as the clustering granularity increases. These additional evaluations demonstrate that the seven-community structure reflects a stable organizational feature of arousal-modulated connectivity

**Reviewer #2 (Public review):**

(1) Arousal effects on BOLD signals and on pupil size can have different delays, so it would be valuable to test lagged relationships (for example, shifting the pupil series forward and backward) to show that the main community structure and lateralization results are not sensitive to an arbitrary temporal alignment.

We agree with you that accounting for the varying delays between BOLD signals and pupillary dynamics is essential for ensuring the robustness of our results. We conducted a comprehensive lagged cross-correlation analysis to address it. Following established frameworks for linking BOLD signals with pupillometry (Yellin et al., 2015; Gonzalez-Castillo et al., 2022; Lloyd et al., 2023), we systematically shifted the pupil time series relative to the fMRI data by -3 TR to +3 TR (-3s to +3s) and evaluated the consistency of the community architecture across these lags using Dice coefficients.

As shown in Figure S5C, these results demonstrate that the core community organization remain stable across the tested range of physiological delays. This stability confirms that our findings are not sensitive to an arbitrary temporal alignment but instead reflect a robust neurobiological phenomenon that persists throughout the physiologically plausible lag window.

(2) Pupil diameter covaries with blinks, eye closure, and other factors that can covary with head motion and physiological noise. The Methods include substantial quality control and denoising, including motion regression and scrubbing, plus exclusions for eye closure.

We appreciate your attention to these potential confounding factors. While we implemented rigorous preprocessing including regressing out confounds on fMRI images, we agree that physiological noise and motion may influenced pupil signals.

To address this, we conducted an additional control analysis where we included head motion (framewise displacement, FD) and the global signal (defined as the mean signal across all gray matter voxels) as covariates when calculating the arousal–tvFC coupling. We then re-evaluated the similarity between the resulting community architecture and our original findings. As shown in Figure S4, the community structure remained stable after controlling for these variables.

Regarding eye closure, we intentionally did not regress this out, as extensive literature demonstrates that eye closure is itself a reliable physiological proxy for arousal levels (Sommer & Golz, 2010; Chang et al., 2016; Gonzalez-Castillo et al., 2022); regressing it out would likely remove the very arousal-related coupling effects we aim to investigate.

(3) The dataset is described in terms of runs retained (for example, 485 resting runs), and runs are treated as observations in clustering after z-scoring across runs. If multiple runs come from the same individuals, the manuscript would benefit from explicitly showing that results replicate at the participant level (for example, community structure stability within participant across runs, and participant-level summary statistics used for inference), rather than relying primarily on pooled run-level patterns.

We fully agree with you that it is essential to demonstrate that the observed hemispheric asymmetries reflect a stable neurobiological principle rather than an artifact of sampling variability or high-dimensional noise. To address this concern, we performed two rigorous validation analyses using 500-iteration resampling schemes, consisting of a split-half reliability test and a participant-level consistency assessment.

First, to ensure our findings do not depend on specific sample compositions, we conducted a split-half reliability test where the dataset was randomly partitioned into two independent subgroups over 500 iterations. As shown in Figure S1A, the community labels maintained high spatial consistency across iterations (as evidenced by the confusion matrix and Dice coefficient distributions), and our original findings—including network-pair community architecture (Fig. S2A), regional affiliation patterns (Fig. S3A-B), and arousal–tvFC coupling lateralization (Fig. S4A-B)—were consistently situated at the center of the iteration distributions.

Second, to account for potential within-participant dependencies in the HCP 7T dataset, we performed a participant-level resampling analysis (N = 139). By randomly selecting a different session for each participant across 500 iterations, we confirmed that the community architecture and hemispheric biases remain robust even under this strict control (Figure S1A, S2B, S3C-D and S4C-D). Collectively, these additional analyses provide strong evidence that the hemispheric lateralization we reported is not a byproduct of sampling bias, but instead represents a stable organizational principle of the arousal-modulated connectome.

(4) Time-resolved connectivity is estimated using a 30-second sliding window and 5 second step. It is reasonable to wonder whether the same conclusions hold with alternative estimators that do not rely on fixed windows. The Discussion acknowledges this limitation, but adding a small robustness analysis would make the paper more definitive.

To ensure that our findings are not artifacts of a specific analytical choice, we performed an exhaustive sensitivity analysis by repeating our entire pipeline across a wide range of window lengths (30s, 35s, 60s, and 90s) and step sizes (1s, 5s, and 10s). We then employed Dice coefficients to quantify the topological similarity between these alternative configurations and our original parameters (30s window, 5s step).

As shown in Figure S3, our results demonstrate high topological consistency, with Dice coefficients for community structures remaining consistently above 0.8 across all tested parameter combinations. Furthermore, the core hemispheric asymmetry patterns were robustly preserved regardless of the specific windowing configuration used. These results provide strong evidence that the arousal-modulated organizational principles we reported are inherent to the data and are stable across a broad range of temporal scales.

**Reviewer #3 (Public review):**

(1) A major limitation of the study is the limited discussion of subcortical regions, which play a central role in arousal regulation according to extensive prior literature. Although the current analyses focus primarily on cortical organization, the authors should include a brief discussion of how their findings relate to subcortical arousal systems.

We completely agree that subcortical structures are pivotal drivers of arousal regulation. While our study primarily utilized a symmetric cortical atlas to ensure a mathematically rigorous assessment of hemispheric lateralization, we recognize that the exclusion of subcortical regions limits the functional interpretation of the observed patterns.

In the revised manuscript, we have added a dedicated discussion part (page 20, lines 412-428) to address this point:

“First, to ensure a mathematically rigorous assessment of hemispheric asymmetry, our analysis was restricted to a symmetric cortical parcellation. Consequently, while we demonstrate that arousal-modulated connectivity follows a structured macroscopic architecture, we did not explicitly analyze the subcortical nuclei hypothesized to drive these patterns. We hypothesize that the presence of these low-dimensional cortical communities reflects coordinated motifs rather than a homogeneous gain modulation, potentially mirroring the differentiated projection patterns of subcortical neuromodulatory systems. For instance, the locus coeruleus–noradrenergic pathway (Chandler et al., 2014; Schwarz & Luo, 2015) and thalamus (Hwang et al., 2017; Shine, 2019; Müller et al., 2020; Shine et al., 2023) possess extensive yet non-uniform projections that may anchor the community-specific and hemispherically asymmetric patterns observed here. “

(2) While sliding window methods can capture temporal changes in functional organization, they have limitations in characterizing moment-to-moment neural fluctuations. In particular, results can be highly sensitive to window length and step size. The manuscript would benefit from (a) a clearer discussion of these methodological limitations, (b) justification for the chosen window length and step size, and (c) a sensitivity analysis demonstrating whether the main findings are robust across different parameter choices.

To ensure that our findings are not artifacts of a specific analytical choice, we performed an exhaustive sensitivity analysis by repeating our entire pipeline across a wide range of window lengths (30s, 35s, 60s, and 90s) and step sizes (1s, 5s, and 10s). We then employed Dice coefficients to quantify the topological similarity between these alternative configurations and our original parameters (30s window, 5s step).

As shown in Figure S5, our results demonstrate high topological consistency, with Dice coefficients for community structures remaining consistently above 0.8 across all tested parameter combinations. Furthermore, the core hemispheric asymmetry patterns were robustly preserved regardless of the specific windowing configuration used. These results provide strong evidence that the arousal-modulated organizational principles we reported are inherent to the data and are stable across a broad range of temporal scales.

(2) The authors use k-means clustering to identify groups of brain regions and refer to these groupings as "communities." However, in general, community detection typically refers to graph-based algorithms that identify modules based on connectivity structure (e.g., modularity maximization). The clusters derived from k-means in feature space are not necessarily equivalent to graph-theoretic communities. The authors should explicitly clarify this distinction and adjust terminology accordingly to avoid conceptual ambiguity.

We agree that the term "community detection" is often specifically associated with graph-based algorithms, such as modularity maximization, which define modules based on topological connectivity. In contrast, our implementation of k-means identifies groupings based on the similarity of arousal–FC coupling patterns within a high-dimensional feature space.

To avoid any conceptual ambiguity or potential confusion, we have explicitly clarified this distinction in the Methods (pages 24-25, lines 533-542) section of the revised manuscript:

“We employed the k-means clustering algorithm (Euclidean distance) to explore a range of cluster solutions from K = 2 to 15. To ensure the stability of the results and avoid local optima, each K was repeated 250 times with random initializations. The optimal number of clusters was determined by evaluating clustering quality and reproducibility (e.g., maximizing silhouette stability). It is important to clarify that "communities" in this context refer to clusters of edges that exhibit similar arousal-modulation motifs within a high-dimensional feature space, rather than topological modules typically derived from graph-theoretic algorithms like modularity maximization. This procedure consistently identified seven distinct communities, each representing a robust, arousal-sensitive connectivity motif that characterizes the large-scale organization of brain-pupil coupling.”

**Recommendations for the authors:**

**Reviewer #1 (Recommendations for the authors):**

(1) To strengthen confidence in the reported hemispheric effects, the authors should provide additional robustness analyses, such as subject-level consistency of lateralization measures, split-half or resampling reliability, and sensitivity to alternative preprocessing or analysis choices. Reporting the distribution of lateralization effects across individuals would help clarify whether the observed asymmetries reflect stable features or group-level averages driven by a subset of connections or participants.

We agree that establishing the individual-level stability of lateralization is essential. We have now provided extensive validation, including split-half reliability tests and participant-level consistency analyses (500 iterations). These results confirm that the reported asymmetries are robust and consistent across the sample. Please refer to Reviewer #1 Weakness2 for the full analysis and associated figures (Figure. S1-S4).

(2) The authors should examine whether arousal-connectivity coupling patterns are robust to plausible temporal delays between pupil diameter and BOLD signals. Lagged or time-shifted analyses would help establish that the findings do not depend on a specific zero-lag assumption.

We agree that validating the coupling between pupil dynamics and the time varying FC is essential. To address this, we conducted a lag sensitivity analysis by shifting the pupil-derived arousal signal within a physiologically plausible range (-3 to +3 TR). The community architecture remains highly consistent across these temporal offsets, showing high spatial correlation and Dice coefficients with our original findings. This stability confirms that the identified organizational motifs are robust and not dependent on a specific zero-lag assumption. For the full details of this validation and the associated figures, please refer to Reviewer #1 Weakness3 and Figure S5 in the Supplementary Material.

(3) Given reliance on a single sliding-window length, the authors should assess how key results vary across different window sizes. Demonstrating stability of the community structure and lateralization patterns across parameter choices would strengthen the methodological foundation of the study.

We have conducted an exhaustive sensitivity analysis across various window lengths (30s, 35s, 60s, 90s) and step sizes (1s, 5s, 10s). The high Dice coefficients (>0.8) confirm that our findings are not dependent on specific windowing choices. Please refer to Reviewer #1 Weakness3 and Figure S5 for the full results.

(4) The justification for the chosen number of connectivity communities would benefit from additional clustering evaluations. Complementary criteria such as measures of compactness and separation, model selection approaches for determining the number of clusters, and stability or reproducibility under resampling would help establish whether the reported community structure is robust rather than method-dependent.

To strengthen the mathematical basis for our partition, we have implemented a multi-metric evaluation and the L-method for objective K selection. These metrics consistently support the seven-community structure. Please refer to our response to Reviewer #1 Weakness5 and Figure S7 for the comprehensive evaluation.

(5) The manuscript would benefit from a clearer discussion of why ultra-high-field imaging was required for the present analyses and whether similar results are expected at standard field strengths. If feasible, validation using lower-field data or reference to existing datasets would substantially enhance generalizability.

We have expanded our discussion to clarify that 7T was instrumental for capturing the subtle, high-frequency arousal-tvFC coupling due to its superior SNR. We also explicitly discuss the potential and limitations of generalizing these findings to 3T datasets. Please refer to our response to Reviewer #1 Weakness2 for the full discussion (page 21, lines 447-456).

(6) The authors should more explicitly report exclusion related to pupil measurements and discuss how missing or noisy pupillometry may affect the applicability of the approach in other datasets or experimental settings.

We agree that transparency in data screening is essential for the reproducibility of our method. In the revised manuscript, we have clarified our quality control pipeline in the quality control section in Methods (page 23, lines 502-510):

“The final analyzed sample for the resting-state consisted of N = 139 healthy participants (mean age = 29.1±3.5 years, 77 female). Runs were excluded if (a) more than 20% of frames exceeded motion thresholds, (b) eye tracking did not cover the full fMRI time series, or (c) more than 90% of samples were classified as eye closure. After applying these criteria, 485 of the initial 723 scans were retained for analysis. The same quality-control pipeline was applied to the movie-watching dataset, yielding 513 usable scans out of the original 725. Detailed information on data retention and run distribution per participant is summarized in Figure S9.”

Furthermore, we have added a discussion regarding how noisy or missing pupillary signals might affect the generalizability of our approach (pages 20-21, lines 437-447):

“Fourth, the generalizability of our approach to external cohorts warrants caution regarding pupillary data integrity. In contexts where high-fidelity eye-tracking is technically demanding—such as in clinical settings involving patients with restricted compliance or in naturalistic fMRI studies—the prevalence of blink artifacts and signal dropouts may bias the estimation of arousal-modulated states. Excessive reliance on data interpolation in such cases could artificially smooth temporal fluctuations, leading to an overestimation of community stability. Future applications should therefore prioritize high-frequency sampling and potentially incorporate multi-modal physiological features (e.g., respiratory or cardiac signals) to cross-validate arousal dynamics when pupillary data is suboptimal (Meissner et al., 2023; Bolt et al., 2025; Weijs et al., 2025).”

(7) The authors should ensure that all data and analysis code necessary to reproduce the results are made publicly available in accordance with eLife policies, including clear documentation of preprocessing steps, parameter choices, and clustering procedures.

All analysis code and the necessary processed data required to reproduce our findings have been made publicly available through https://github.com/kongxy6478/Arousal-modulates-functional-connectivity. This repository includes documented pipelines for pupillometry cleaning and fMRI denoising, alongside the core Python scripts used for sliding-window connectivity calculation, k-means clustering, and hemispheric lateralization analysis.

**Reviewer #2 (Recommendations for the authors):**

(1) Add a lag sensitivity analysis between pupil-derived arousal and time-resolved connectivity, and report whether the seven community structure and key lateralization findings are stable across a plausible lag range.

We agree that validating the coupling between pupil dynamics and the time varying FC is essential. To address this, we conducted a lag sensitivity analysis by shifting the pupil-derived arousal signal within a physiologically plausible range (-3 to +3 TR). The community architecture remains highly consistent across these temporal offsets, showing high spatial correlation and Dice coefficients with our original findings. This stability confirms that the identified organizational motifs are robust and not dependent on a specific zero-lag assumption. For the full details of this validation and the associated figures, please refer to Reviewer #1 Weakness3 and Figure S5 in the Supplementary Material.

(2) Quantify and report the extent to which residual head motion, blink rate, eye closure segments, and global signal changes explain arousal connectivity coupling, for example, via partial correlation or regression controls, and show that key effects persist.

We agree that it is essential to demonstrate that the observed arousal-connectivity coupling is not driven by non-specific physiological or motion-related artifacts. As requested, we have quantified the influence of head motion (FD) and global signal on our primary results. By implementing partial correlation analyses, we confirmed that the identified arousal-modulated community structures persist even after strictly controlling for these variables. These results indicate that the arousal-tvFC coupling we report reflects a specific neuro-arousal process rather than a byproduct of motion or systemic physiological fluctuations. For the detailed quantitative results and control analysis figures, please refer to our response to Reviewer #2 Weakness3 and Figure S6 in the Supplementary Material.

(3) Add participant-level validation: demonstrate that community profiles and lateralization signatures are consistent within participants across runs, and consider participant-level statistical summaries rather than treating all runs as independent observations.

We agree that demonstrating participant-level consistency is vital. In response, we performed two rigorous 500-iteration resampling schemes: a split-half reliability test and a participant-level consistency assessment (N = 139). These analyses, which involved randomly partitioning the sample and selecting single sessions per participant, confirm that our community architecture and hemispheric biases are remarkably stable and not driven by sampling variability or high-dimensional noise. For a comprehensive description of these validations and the associated statistical distributions, please refer to our detailed response to Reviewer #2 Weakness3 and Figures S1–S4.

(4) Provide an alternative dynamic connectivity estimator robustness check, or at a minimum, vary the window length and step size to show stability of the primary conclusions.

We have conducted an exhaustive sensitivity analysis across various window lengths (30s, 35s, 60s, 90s) and step sizes (1s, 5s, 10s). The high Dice coefficients (>0.8) confirm that our findings are not dependent on specific windowing choices. Please refer to Reviewer #1 Weakness3 and Figure S5 for the full results.

(5) Consider validating the seven community solutions with at least one additional unsupervised approach, and report agreement with the main k-means solution.

We agree that validating the clustering scheme is essential. To this end, we implemented a multi-criteria evaluation (including Davies-Bouldin and Silhouette indices) and utilized the L-method (Salvador & Chan, 2004) to mathematically confirm K=7 as the optimal granularity (Figure S7A–B). Furthermore, we verified that the core topological features and hemispheric asymmetries remain robustly consistent across a range of granularities from K=5 to 9 (Figure S7C). These analyses demonstrate that our findings are not dependent on a specific K or subjective bias. For the full quantitative evaluation and stability maps, please refer to our response to Reviewer #2 Weakness5 and Figure S7.

(6) State explicitly, early in Results, what the main inferential unit is (run or participant) for each key analysis, and clarify how repeated runs per participant are handled.

We agree that defining the inferential unit is critical for methodological clarity. In the revised manuscript, we have explicitly stated at the beginning of the Results section (page 5, lines 113-116):

“While our primary inferential analyses were conducted at the run level to leverage the high-density sampling of the HCP 7T dataset, we further validated the robustness of these findings using participant-level statistical summaries and resampling to account for within-participant dependencies (see Figure. S1-S2 in Supplementary Materia).”

Specifically, all key findings—including community architecture and hemispheric asymmetries—were validated using participant-level statistics and resampling schemes (N = 139) to ensure that the results are not biased by within-participant dependencies.

(7) When introducing the integration and segregation indices, add a brief intuitive explanation of what a positive or negative value means in plain language before the equations.

We thank the reviewer for this suggestion to improve the accessibility of our methods. We have added brief, intuitive explanations for both indices in the Methods section (pages 26-27, lines 569-582):

“The integration index provides a measure of the overall hemispheric dominance of arousal-modulated connections. A positive value indicates that arousal-related edges are preferentially concentrated in the left hemisphere (including its internal and outgoing connections) compared to the right.” and “The segregation index assesses whether arousal preferentially modulates local, intra-hemispheric communication versus long-range, inter-hemispheric communication. A positive value reflects a "segregated" left-hemisphere bias, where arousal strengthens within-hemisphere connections more than it strengthens across-hemisphere communication for that same hemisphere. “

(8) In the Discussion, separate claims into "what we show" versus "what we hypothesize," especially when connecting findings to neuromodulatory pathways.

In the revised manuscript, we have carefully separated our direct empirical findings from our mechanistic hypotheses. we have utilized more cautious and speculative language (e.g., "suggesting a potential role of," "may be mediated by," and "we hypothesize that”) (page 17, lines 352-358):

“Specifically, we show the presence of low-dimensional, reproducible communities suggests that arousal modulates the connectome through coordinated motifs rather than homogeneous gain modulation. We hypothesize that this structured macroscopic architecture reflects the differentiated projection patterns of subcortical neuromodulatory systems, such as the locus coeruleus–noradrenergic pathway (Aston-Jones & Cohen, 2005; Jordan, 2024) and thalamus (Magnin et al., 2010; Lewis et al., 2015; Liu et al., 2018)”

(9) Provide a clear participant-level summary (number of participants contributing to the retained runs, demographics if available, and distribution of runs per participant), alongside the reported run counts retained after quality control.

We agree that clear reporting of participant-level data is essential. In the revised Methods section, we have added a detailed summary of participant demographics (age and sex) and clarified the sample composition (page 23, lines 502-503):

“The final analyzed sample for the resting-state consisted of N = 139 healthy participants (mean age = 29.1±3.5 years, 77 female).”

Furthermore, to provide a transparent view of the data retained after quality control, we have included Figure S9 to illustrate the distribution of valid runs per participant. This visualization confirms the amount of data contributing to our group-level inferences and accounts for exclusions due to motion or pupillary signal quality.

(10) Report the robustness of results to reasonable changes in pupil preprocessing choices (for example, smoothing parameters or interpolation rules), since pupil diameter is the key arousal index.

We agree that the robustness of pupil-derived arousal estimates is fundamental to our findings. To address this, we conducted an extensive validation analysis by comparing our original pupil preprocessing pipeline against 18 alternative combinations of parameters. These variations included different smoothing window sizes (100 ms, 200 ms, and 500 ms), interpolation methods (linear vs. cubic spline), and blink buffer durations (25 ms, 50 ms, and 100 ms). As shown in Figure S8, the pupil diameter time courses derived from these diverse pipelines remained highly correlated with our original estimates (all above 0.65). This demonstrates that our arousal-modulated connectivity results are remarkably robust to reasonable changes in pupil preprocessing choices.

**Reviewer #3 (Recommendations for the authors):**

I have two additional minor comments:

(1) Given the overall goal of this study to identify large-scale brain communities or clusters underlying arousal, the results may be sensitive to the choice of cortical parcellation. The authors should consider:

(a) including analyses using additional parcellation schemes, or

(b) discussing how the current findings might depend on the chosen parcellation and the implications for robustness and generalizability.

We have addressed this by adding a dedicated point in the Discussion (page 21, lines 456-465):

“Sixth, our findings were derived using a single high-resolution cortical parcellation. While the specific choice of atlas can influence fine-grained regional connectivity, it is important to note that our primary conclusions—such as hemispheric asymmetries and community-level preferences—were identified and interpreted at the macroscopic network and system level. By aggregating signals across broad functional systems, this approach likely mitigates the dependency on precise regional boundary definitions. Nevertheless, future studies employing alternative parcellation schemes would be valuable to further confirm that these organizational principles are not specific to the current atlas but represent a generalizable feature of the arousal-modulated connectome.”

(2) Some key details, such as the number of participants included in the study, as well as basic demographic information, are not reported.

We apologize for this omission. In the revised Methods section, we have now included a detailed summary of the participant demographics, including the final sample size (N = 139), age, and sex distribution (page 23, lines 502-503):

“The final analyzed sample for the resting-state consisted of N = 139 healthy participants (mean age = 29.1±3.5 years, 77 female)”

Furthermore, to ensure full transparency regarding data retention, we have added a new figure (Figure S9) illustrating the distribution of valid fMRI runs per participant following our quality-control procedures. We believe these additions provide a clear and complete overview of the study sample.

Reference

Aston-Jones, G., & Cohen, J. D. (2005). AN INTEGRATIVE THEORY OF LOCUS COERULEUS-NOREPINEPHRINE FUNCTION: Adaptive Gain and Optimal Performance. In Annual Review of Neuroscience (Vol. 28, Issue Volume 28, 2005, pp. 403–450). Annual Reviews. https://doi.org/10.1146/annurev.neuro.28.061604.135709

Bolt, T., Wang, S., Nomi, J. S., Setton, R., Gold, B. P., deB.Frederick, B., Yeo, B. T. T., Chen, J. J., Picchioni, D., Duyn, J. H., Spreng, R. N., Keilholz, S. D., Uddin, L. Q., & Chang, C. (2025). Autonomic physiological coupling of the global fMRI signal. Nature Neuroscience, 28(6), 1327–1335. https://doi.org/10.1038/s41593-025-01945-y

Chandler, D. J., Gao, W.-J., & Waterhouse, B. D. (2014). Heterogeneous organization of the locus coeruleus projections to prefrontal and motor cortices. Proceedings of the National Academy of Sciences, 111(18), 6816–6821. https://doi.org/10.1073/pnas.1320827111

Chang, C., Leopold, D. A., Schölvinck, M. L., Mandelkow, H., Picchioni, D., Liu, X., Ye, F. Q., Turchi, J. N., & Duyn, J. H. (2016). Tracking brain arousal fluctuations with fMRI. Proceedings of the National Academy of Sciences, 113(16), 4518–4523. https://doi.org/10/f8ktgg

Gonzalez-Castillo, J., Fernandez, I. S., Handwerker, D. A., & Bandettini, P. A. (2022). Ultra-slow fMRI fluctuations in the fourth ventricle as a marker of drowsiness. NeuroImage, 259, 119424. https://doi.org/10.1016/j.neuroimage.2022.119424

Hwang, K., Bertolero, M. A., Liu, W. B., & D’Esposito, M. (2017). The Human Thalamus Is an Integrative Hub for Functional Brain Networks. The Journal of Neuroscience, 37(23), 5594–5607. https://doi.org/10.1523/JNEUROSCI.0067-17.2017

Jordan, R. (2024). The locus coeruleus as a global model failure system. Trends in Neurosciences, 47(2), 92–105. https://doi.org/10.1016/j.tins.2023.11.006

Lewis, L. D., Voigts, J., Flores, F. J., Schmitt, L. I., Wilson, M. A., Halassa, M. M., & Brown, E. N. (2015). Thalamic reticular nucleus induces fast and local modulation of arousal state. eLife, 4, e08760. https://doi.org/10.7554/eLife.08760

Liu, X., De Zwart, J. A., Schölvinck, M. L., Chang, C., Ye, F. Q., Leopold, D. A., & Duyn, J. H. (2018). Subcortical evidence for a contribution of arousal to fMRI studies of brain activity. Nature Communications, 9(1), 395. https://doi.org/10.1038/s41467-017-02815-3

Lloyd, B., De Voogd, L. D., Mäki-Marttunen, V., & Nieuwenhuis, S. (2023). Pupil size reflects activation of subcortical ascending arousal system nuclei during rest. eLife, 12, e84822. https://doi.org/10.7554/eLife.84822

Magnin, M., Rey, M., Bastuji, H., Guillemant, P., Mauguière, F., & Garcia-Larrea, L. (2010). Thalamic deactivation at sleep onset precedes that of the cerebral cortex in humans. Proceedings of the National Academy of Sciences, 107(8), 3829–3833. https://doi.org/10.1073/pnas.0909710107

Meissner, S. N., Bächinger, M., Kikkert, S., Imhof, J., Missura, S., Carro Dominguez, M., & Wenderoth, N. (2023). Self-regulating arousal via pupil-based biofeedback. Nature Human Behaviour, 8(1), 43–62. https://doi.org/10.1038/s41562-023-01729-z

Müller, E. J., Munn, B., Hearne, L. J., Smith, J. B., Fulcher, B., Arnatkevičiūtė, A., Lurie, D. J., Cocchi, L., & Shine, J. M. (2020). Core and matrix thalamic sub-populations relate to spatio-temporal cortical connectivity gradients. NeuroImage, 222, 117224. https://doi.org/10.1016/j.neuroimage.2020.117224

Salvador, S., & Chan, P. (2004). Determining the number of clusters/segments in hierarchical clustering/segmentation algorithms. 16th IEEE International Conference on Tools with Artificial Intelligence, 576–584. https://doi.org/10.1109/ICTAI.2004.50

Schwarz, L. A., & Luo, L. (2015). Organization of the Locus Coeruleus-Norepinephrine System. Current Biology, 25(21), R1051–R1056. https://doi.org/10.1016/j.cub.2015.09.039

Shine, J. M. (2019). Neuromodulatory Influences on Integration and Segregation in the Brain. Trends in Cognitive Sciences, 23(7), 572–583. https://doi.org/10.1016/j.tics.2019.04.002

Shine, J. M., Lewis, L. D., Garrett, D. D., & Hwang, K. (2023). The impact of the human thalamus on brain-wide information processing. Nature Reviews Neuroscience, 24(7), 416–430. https://doi.org/10.1038/s41583-023-00701-0

Sommer, D., & Golz, M. (2010). Evaluation of PERCLOS based current fatigue monitoring technologies. 2010 Annual International Conference of the IEEE Engineering in Medicine and Biology, 4456–4459. https://doi.org/10.1109/IEMBS.2010.5625960

Weijs, M. L., Missura, S., Potok-Szybińska, W., Bächinger, M., Badii, B., Carro-Domínguez, M., Wenderoth, N., & Meissner, S. N. (2025). Modulating cortical excitability and cortical arousal by pupil self-regulation. Nature Communications, 16(1), 4552. https://doi.org/10.1038/s41467-025-59837-5

Yellin, D., Berkovich-Ohana, A., & Malach, R. (2015). Coupling between pupil fluctuations and resting-state fMRI uncovers a slow build-up of antagonistic responses in the human cortex. NeuroImage, 106, 414–427. https://doi.org/10.1016/j.neuroimage.2014.11.034
